# Continuous year-round isolation of giant viruses from brackish shoreline soils

**DOI:** 10.3389/fmicb.2024.1402690

**Published:** 2024-05-02

**Authors:** Motohiro Akashi, Masaharu Takemura, Seiichi Suzuki

**Affiliations:** ^1^Department of Science and Technology, Faculty of Science and Technology, Seikei University, Tokyo, Japan; ^2^Institute of Arts and Sciences, Tokyo University of Science, Tokyo, Japan

**Keywords:** NCLDV, giant virus, microbial ecology, brackish water, soil

## Abstract

Giant viruses, categorized under *Nucleocytoviricota,* are believed to exist ubiquitously in natural environments. However, comprehensive reports on isolated giant viruses remain scarce, with limited information available on unrecoverable strains, viral proliferation sites, and natural hosts. Previously, the author highlighted *Pandoravirus hades*, *Pandoravirus persephone*, and *Mimivirus* sp. styx, isolated from brackish water soil, as potential hotspots for giant virus multiplication. This study presents findings from nearly a year of monthly sampling within the same brackish water region after isolating the three aforementioned strains. This report details the recurrent isolation of a wide range of giant viruses. Each month, four soil samples were randomly collected from an approximately 5 × 10 m plot, comprising three soil samples and one water sample containing sediment from the riverbed. *Acanthamoeba castellanii* was used as a host for virus isolation. These efforts consistently yielded at least one viral species per month, culminating in a total of 55 giant virus isolates. The most frequently isolated species was *Mimiviridae* (24 isolates), followed by *Marseilleviridae* (23 isolates), *Pandoravirus* (6 isolates), and singular isolates of *Pithovirus* and *Cedratvirus*. Notably, viruses were not consistently isolated from any of the four samples every month, with certain sites yielding no viruses. Cluster analysis based on isolate numbers revealed that soil samples from May and water and sediment samples from January produced the highest number of viral strains. These findings underscore brackish coastal soil as a significant site for isolating numerous giant viruses, highlighting the non-uniform distribution along coastlines.

## 1 Introduction

The habitats in which *Nucleocytoviricota*, also known as nucleocytoplasmic large DNA viruses (NCLDVs) or giant viruses, exhibit considerable diversity, suggesting their presumed ubiquity in natural environments. *Mimivirus*, renowned for bringing giant viruses to the scientific forefront owing to its atypical characteristics, was originally discovered in a water storage tower in France (La Scola et al., 2003). Subsequent isolations of giant viruses have occurred from various locales, including Siberian permafrost, marine sediments, alkaline lakes, and hot spring water puddles (Legendre et al., 2014; Abrahão et al., 2018; Yoshikawa et al., 2019). The extraction and analysis of soil metagenomic samples from forested areas have played a pivotal role in the reconstruction of numerous giant viral genomes, underscoring the significance of soil as a reservoir for these entities (Schulz et al., 2018).

In the case of *Pandoravirus*, viral isolation sources manifest remarkable heterogeneity, spanning marine sediments, irrigation canals, swamp water, and brackish soils (Philippe et al., 2013; Akashi and Takemura, 2019; Pereira Andrade et al., 2019). Investigations employing metabarcoding techniques on *Mimiviridae* viruses sampled from geographically distant aquatic environments (ranging from 74 to 1,765 km apart) have revealed the presence of closely related viruses in disparate locations, suggesting the potential global circulation of giant viruses (Li et al., 2019).

Despite their viral nature, the multiplication strategy of giant viruses involves infecting hosts dispersed in the natural environment. However, as of 2024, the reliable identification of natural hosts for giant viruses remains elusive. Nonetheless, genomic reconstruction and analysis of NCLDVs and scrutiny of virus-host horizontal transmission through global metagenomic datasets suggest a broad association between giant viruses and diverse eukaryotes. The prevalent strategy of host reprogramming, facilitated by viral genes, has emerged as a common characteristic across NCLDVs (Schulz et al., 2020).

The Niemeyer virus was recovered from the Pampulha Lagoon in Brazil, although its presence was discerned in only one of a total of 15 samples procured along the lake’s periphery (Boratto et al., 2015). A similar pattern was observed for Kroon virus, a member of *Mimivirus*, which was isolated from Urban Lake in Lagoa Santa, Brazil. Water samples were collected from 14 distinct locations around the lake periphery, and Kroon virus was isolated from only one of these samples (Boratto et al., 2018). Moreover, mimiviruses and a marseillevirus were successfully isolated from four soil samples originating from nine river basins in Malaysia. The detection of mimivirus and marseillevirus DNA in soil samples was observed in eight sites, and variations in viral abundance were observed across different sampling locations (Tan et al., 2018). These findings collectively underscore the global distribution of giant viruses; however, their presence is notably heterogeneous, spanning environments across several square kilometers.

Extensive studies of giant viruses indicate their global prevalence and contextual dependence on environmental factors. In our exploration and identification of specific growth sites, or “hotspots,” for giant viruses within their natural hosts and ecosystems, we initially posed a fundamental inquiry: “Can giant viruses be consistently isolated from identical locations?” Unfortunately, there remains a conspicuous absence of comprehensive, long-term reproducible studies to ascertain the recurrent isolation of giant viruses from the same sites. The author’s previous endeavor to isolate giant viruses from soil collected from a brackish area of the Arakawa River in Tokyo, Japan, proved successful, resulting in the isolation of two pandoraviruses and one mimivirus from a minute soil sample (Akashi and Takemura, 2019). This study aimed to isolate giant viruses from soil, sediment, and water samples obtained from a brackish water shoreline in a reproducible manner. Since it was unclear at the beginning of the study from where and how many giant viruses could be isolated, we hypothesized that the sampling site from which giant viruses had previously been isolated, would serve as a suitable site for this study. Due to the paucity of confirmed sites for virus isolation, as well as the burden of virus isolation operations, we did not consider any sites other than this one for the purposes of this study.

## 2 Materials and methods

### 2.1 Sampling, virus isolation, and propagation

A systematic monthly collection of soil samples was undertaken over the course of approximately one year within a designated area of the riverbed (approximately 5 m × 10 m). Subsequent isolation experiments involving giant viruses and an amoeba (*Acanthamoeba castellanii*) consistently yielded positive results, confirming the monthly reproducibility of giant virus isolation from the same environmental context. However, in certain instances, viruses were not isolated concurrently from the samples obtained simultaneously, implying an uneven distribution of giant viruses within a local coastal region of approximately 5 × 10 m.

Soil and water specimens were systematically collected from May 2019 to March 2020 along the sandy expanse (35°42′39.2″N, 139°50′54.7″E) beneath the Hirai Bridge in Arakawa, Tokyo. Sampling was conducted during daylight hours, specifically avoiding rain, and at low tide. Each sampling event involved the random selection of three soil samples, which were then collected in 50 mL centrifuge tubes. River water was concomitantly procured in 50 mL centrifuge tubes using a 10 mL pipette, with sediment from the riverbed being perturbed during the collection process.

The procedure for seeding environmental samples into the amoebae commenced with the collection of approximately 1 g of soil in a 1.5 mL tube, followed by vigorous vortexing with 1 mL of sterile water. The supernatant was amalgamated with PYG medium and amoeba (*Acanthamoeba castellanii*), seeded into 96-well plates, and incubated at 26°C for subsequent observation. The PYG medium composition comprised 4 mM CaCl_2_, 4 mM MgSO_4_-7H_2_O, 2.5 mM Na_2_HPO_4_-7H_2_O, 2.5 mM KH_2_PO_4_, 0.1% [w/v] sodium citrate-2H_2_O, 0.05 mM Fe(NH_4_)_2_(SO_4_)_2_-6H_2_O, 100 mM glucose, pH 6.5. Wells displaying cytopathic effects (CPE) were chosen, and the culture medium was extracted and purified from the viral candidates by stepwise dilution in PYG medium containing amoeba. The virus was subsequently co-cultivated with the amoeba in a 25 cm^2^ cell culture flask to facilitate growth.

**Figure 1:**
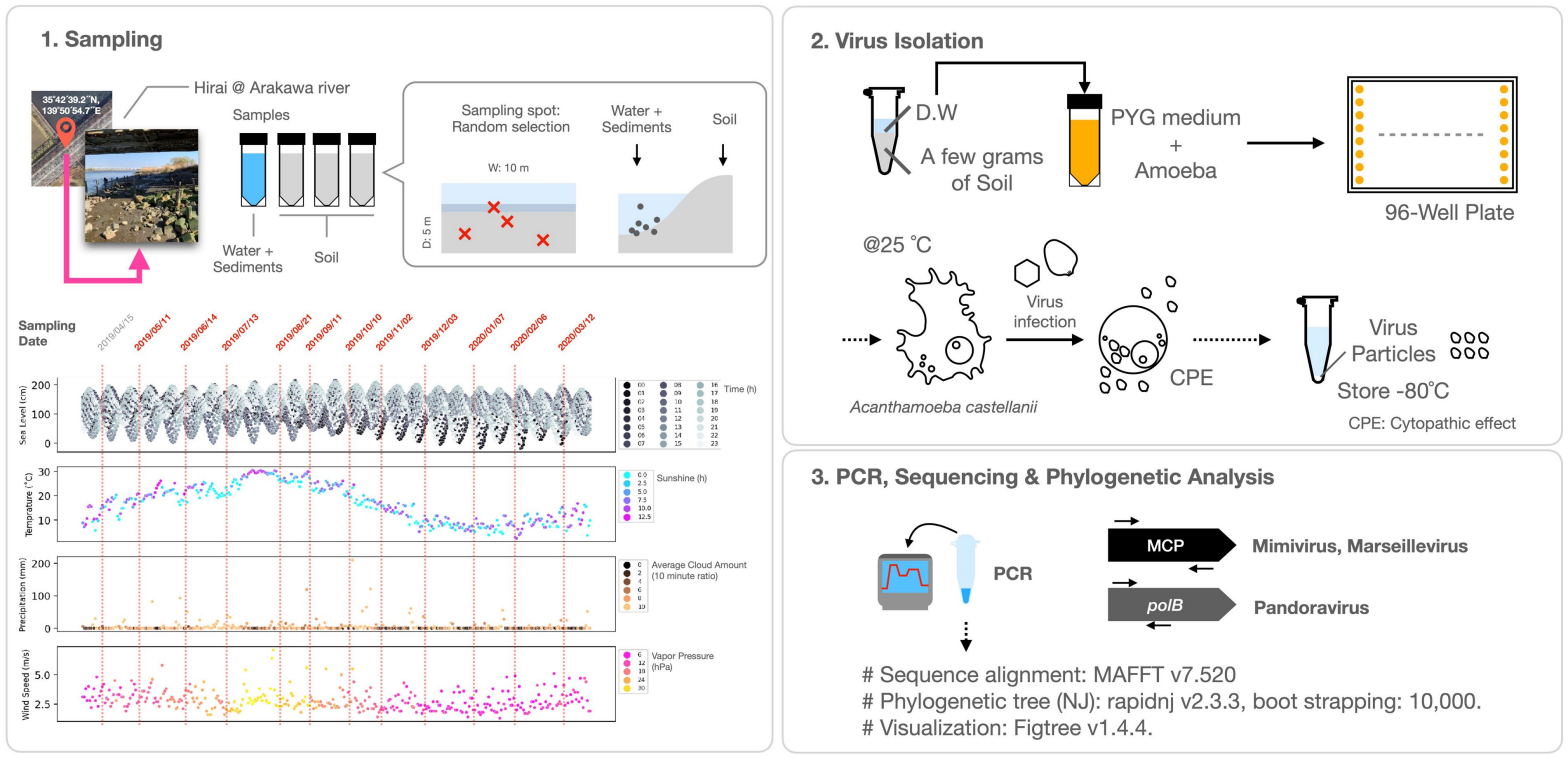
Experimental procedures. The lower left panel of the figure depicts the sampling dates and meteorological data for Tokyo from April 2019 to March 2020, including water level, temperature, sunshine hours, precipitation, cloud cover, wind speed, and vapor pressure. The initial sampling date and time (April 15, 2019) aligns with the Pandoravirus and Mimivirus collection as documented by Akashi and Takemura (2019).

Following growth, the culture medium was centrifuged at 500×*g* for 1 min and the supernatant was collected and subjected to further centrifugation at 8,000×*g* for 35 min to precipitate and gather the virus. The resultant virus solution was preserved at −80°C and subsequently utilized for virus identification through Polymerase Chain Reaction (PCR) analysis (Figure 1).

### 2.2 Virus identification through PCR

Giant virus typing was conducted by PCR using the Tks Gflex DNA Polymerase (Takara Bio, Inc., Tokyo, Japan), and capillary sequencing. For the detection of *Marseilleviridae*, primers targeting the MCP gene (For: 5′-STMYBDTKGAGAGTAATGACTTCTG-3′, Rev.: 5′-RRACTCGGAAGATRTGYTGG-3′) were utilized, as reported previously (Aoki et al., 2019). *Mimivirus* detection involved the use of custom-designed primers amplifying the MCP gene (For: 5′-ATGCTTGGTGAYGTWCCYGAACTWAC-3′, Rev.: 5′-RCTMATSATATTCTGAGAACATTGTAATTRAYMGC-3′). Additionally, primers were employed for the confirmation of the intein on *polB* of *Mimivirus* (For: 5´-GAGACGGATCATGTGGTTCCT-3′, Rev.: 5′-TGGCAGCCCTTTGACACTTC-3′). For the detection of *Pandoravirus*, primers amplifying a partial sequence of the *polB* gene (For: 5′-CCAAACCTCGCACCACTTTG-3′, Rev.: 5′-TCGAGCGTGTACGATTCGAG-3′), as previously reported (Akashi and Takemura, 2019), were used.

The PCR conditions for *Marseillevirus* and *Mimivirus* were as follows: 94°C for 1 min, {98°C for 10 s, 55°C for 15 s, 68°C for 1 min} repeated for 35 cycles, and a final extension at 68°C for 7 min, followed by an infinite hold at 8°C. Viral solutions were utilized as templates in these PCR procedures.

The PCR conditions for *Pandoravirus* were: 94°C for 1 min, {98°C for 10 s, 55°C for 15 s, 68°C for 30 s} repeated for 5 cycles, followed by {98°C for 10 s, 60°C for 15 s, 68°C for 30 s} repeated for 35 cycles, and a final extension at 68°C for 7 min, with an infinite hold at 8°C.

Template DNA was extracted using the standard NucleoSpin Tissue XS protocol (Macherey-Nagel GmbH and Co. KG, Germany). In instances of multibanded PCR products, gel purification was performed using NucleoSpin Gel and PCR Clean-up (Macherey-Nagel), followed by secondary PCR amplification. Only products with single bands were used for subsequent analyses.

PCR for *Pithovirus* and *Cedratvirus* employed the pandoravirus *polB* primers (For: 5´-CCAAACCTCGCACCACTTTG-3′, Rev.: 5´-CTCGGTCCACACCTCGATCG-3′), as previously described (Akashi and Takemura, 2019). PCR conditions for Pithovirus were as follows: 94°C for 1 min, {98°C for 10 s, 24.3°C for 15 s, 68°C for 15 s} repeated for 40 cycles, with an infinite hold at 8°C. For *Cedratvirus*, PCR conditions were 94°C for 1 min, {98°C for 10 s, 11.6°C for 15 s, 68°C for 15 s} repeated for 40 cycles, with an infinite hold at 8°C.

The primers used for PCR were also employed for capillary sequencing, and both the forward and reverse sequences were deciphered. Sequence analysis was conducted using FASMAC Co. The PCR cycling parameters and the FASTA file containing the deciphered sequences are available in Supplementary materials (Supplementary File S1).

### 2.3 Molecular phylogenetic analysis

Molecular phylogenetic analysis was performed using the following procedure. The sequences were aligned using MAFFT (v7.520) with specific options (—genafpair —maxiterate 1,000). Subsequently, Neighbor-Joining (NJ) trees were generated using rapidnj v2.3.3, with 10,000 bootstrap replicates, and the resulting trees were visualized using FigTree v1.4.4. Sequences derived from known viruses used for molecular phylogenetic analysis are documented in the Supplementary File S2. The classification of giant viruses adhered to the guidelines established by the International Committee on Taxonomy of Viruses (ICTV), as of January 2024, and is accessible at https://ictv.global/. In instances where specific classifications were not available at that time, commonly used names were employed.

### 2.4 Visualization of monthly virus isolation counts and environmental information

Python 3.8 was used as the programming platform for visualizing both viral isolate counts and meteorological data. Cluster analysis was conducted using seaborn.clustermap, a function in the Python library. Cluster maps were generated using z-score normalized values for each virus and sample type (i.e., soil and water). Meteorological data pertinent to Tokyo, including temperature, vapor pressure, water level, precipitation, wind speed, cloud cover, and sunshine, were procured from the Japan Meteorological Agency.^1^

## 3 Results

### 3.1 Analysis of phylogenetic diversity of giant viruses isolated from soils in and around brackish water areas

**Figure 2:**
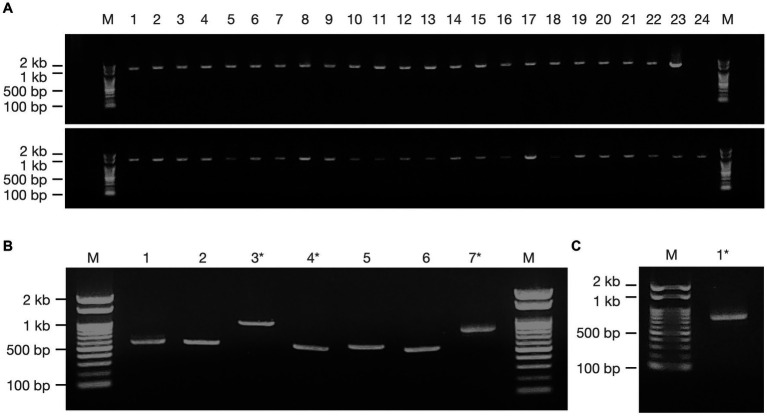
Amplification of giant viral gene fragments by PCR. PCR fragments of the isolated viruses employed for sequencing analysis are illustrated, comprising **(A)** Fragments from *Marseilleviridae* (upper panel) and *Mimiviridae* (lower panel), **(B)** *Pandoravirus* (1–6) and *Cedratvirus* (7), and **(C)** *Pithovirus*. Marker (M): Nippon Gene, Gene ladder 100, 5 μL. * denotes samples obtained by cut-out purification.

A total of 55 strains of giant viruses were isolated from water samples, including river soil and surface sediments of the riverbed along the Arakawa River in Tokyo, through monthly sampling over approximately one year (Figure 2). These strains comprised 23 isolates of *Marseilleviridae*, 24 isolates of *Mimiviridae*, and six isolates of *Pandoravirus* (Figures 2A,B). Additionally, two previously unreported isolates, one each of *Cedratvirus* and *Pithovirus*, were obtained, marking their first isolation in Japan (Figures 2B,C).

**Figure 3:**
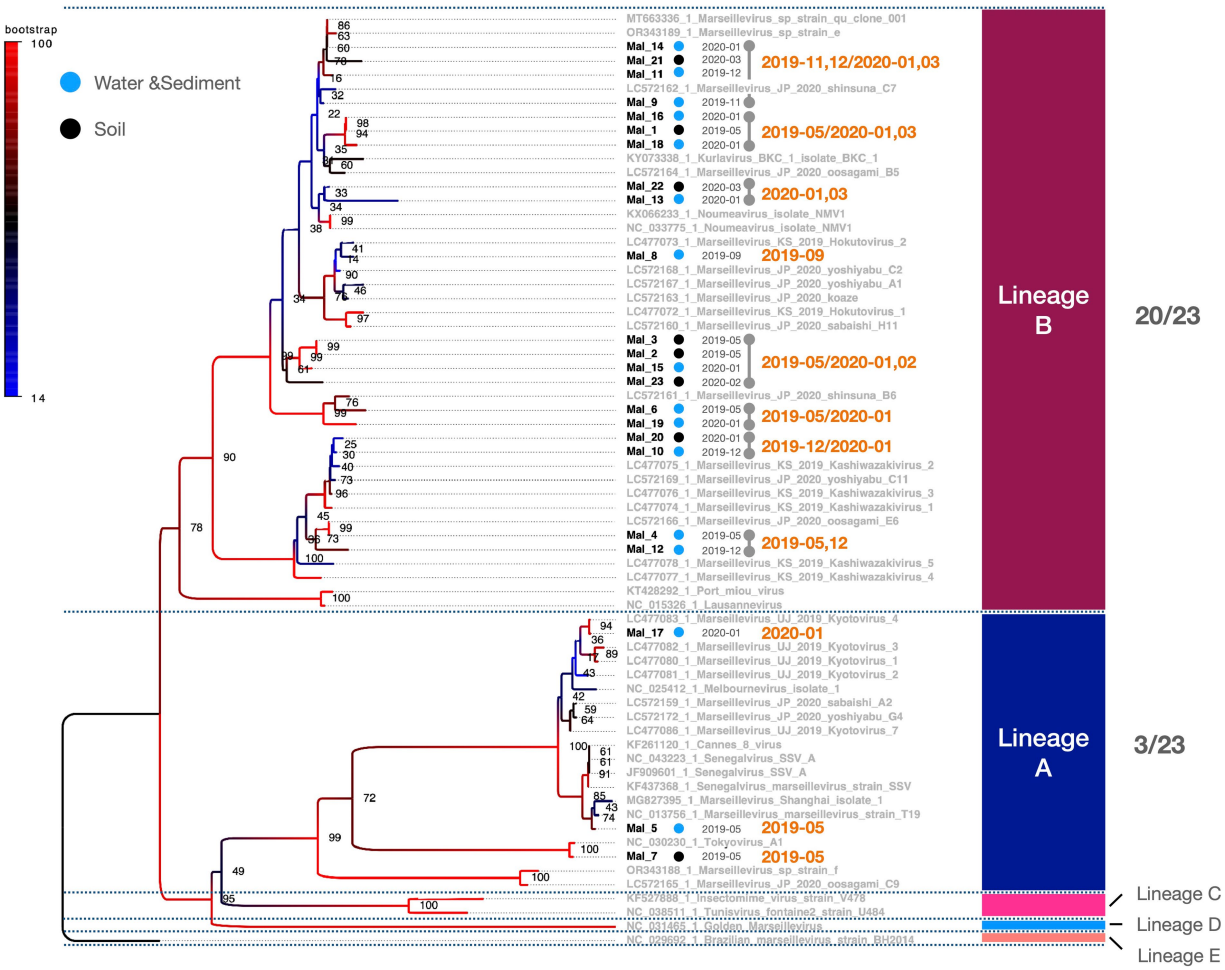
Phylogenetic classification of *Marseilleviridae.* The molecular phylogenetic tree of *Marseilleviridae*, based on 1,394 bp alignment data using MCP, presents OTUs in black (strains isolated in this study) and OTUs in grey (known strains). Black circles denote isolates from soil samples, whereas blue circles represent isolates from water and sediment. The date adjacent to the isolate indicates the month of isolation.

**Figure 4:**
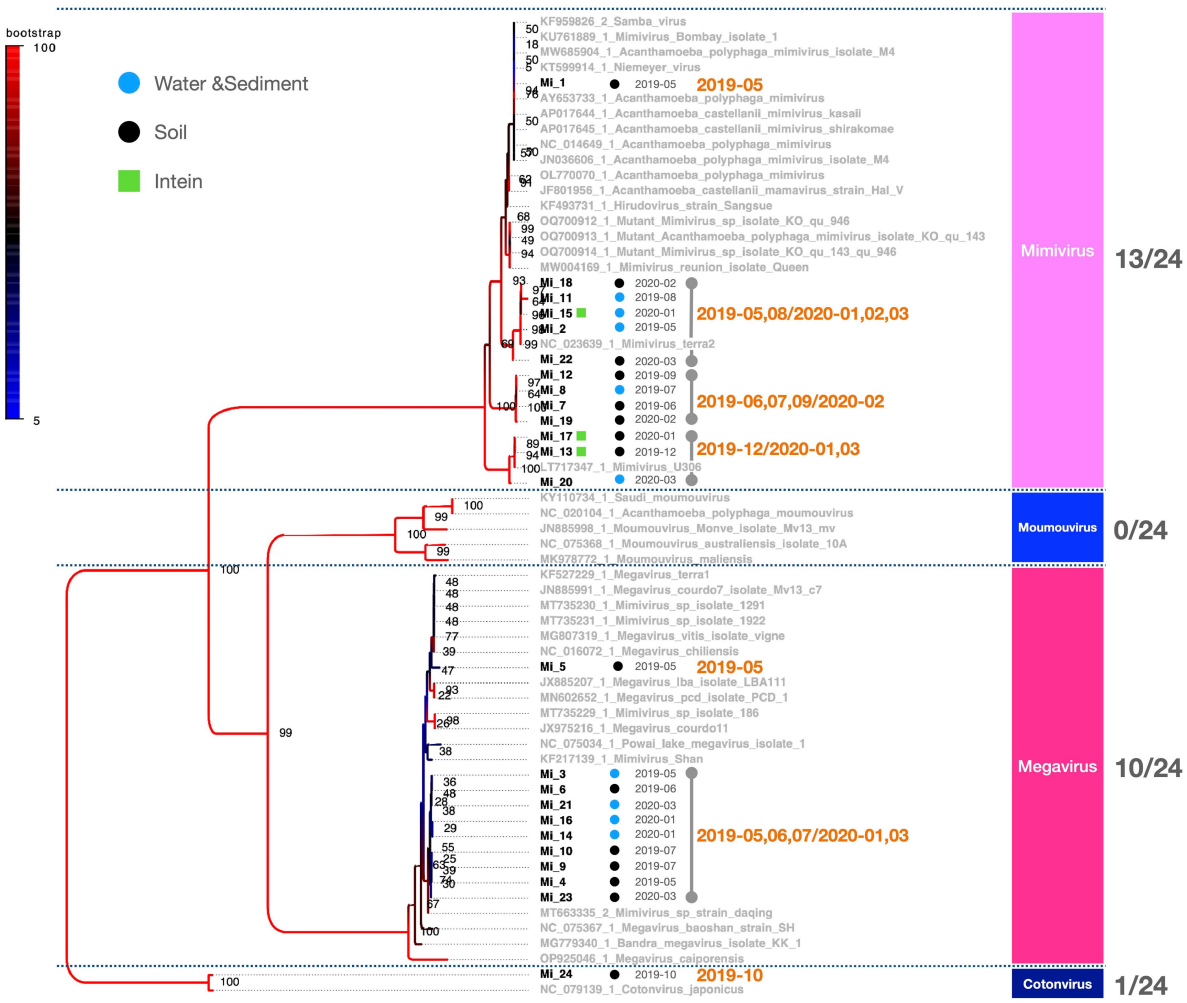
Phylogenetic classification results for *Mimiviridae.* The molecular phylogenetic tree of *Mimiviridae*, based on 1,216 bp alignment data using MCP, illustrates OTUs in black (strains isolated in this study) and OTUs in grey (known strains). Black circles indicate isolates from soil samples and blue circles represent isolates from water and sediment. The date adjacent to the isolate indicates the month of isolation.

PCR fragments of approximately 1.2 kb were successfully obtained from both the marseilleviruses and mimiviruses (Figure 2A). Molecular phylogenetic analysis utilizing the acquired sequence information revealed that among the 23 *Marseilleviridae* strains, three belonged to Lineage A whereas the remaining 20 were of Lineage B (Figure 3). Out of the 24 *Mimiviridae* strains, 13 were classified within the *Mimivirus* family, 10 were categorized as *Megavirus* strains, and one was designated as a *Cotonvirus*. A primer set targeting the intein-containing region *polB* of mimivirus yielded amplification in only three strains: Mi_13, Mi_15, and Mi_17 (Figure 4). Notably, both marseilleviruses and mimiviruses exhibited heterogeneity in the timing and source of isolation, with closely related strains spanning multiple months within both sub-phylogenetic groups (Figures 3, 4).

**Figure 5:**
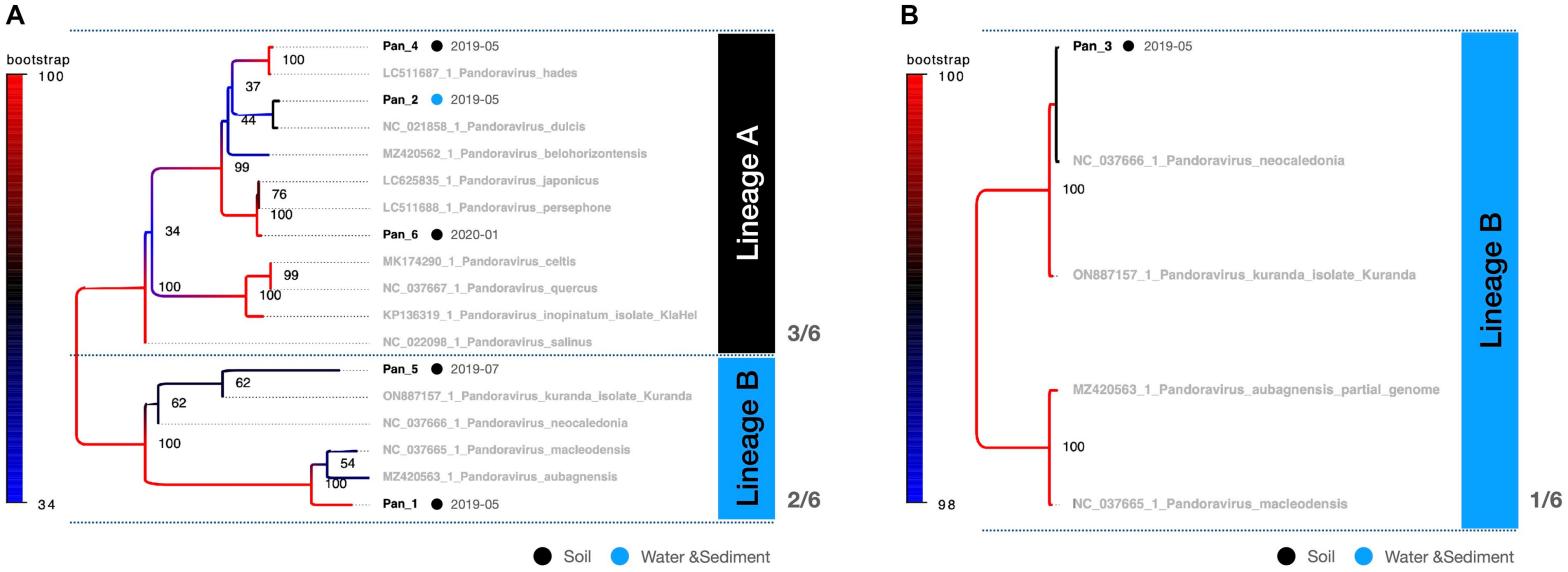
Phylogenetic classification results for *Pandoravirus.* The molecular phylogenetic tree of *Pandoravirus* displays black OTUs (strains isolated in this study) and grey OTUs (known strains). Black circles represent isolates from soil samples, whereas blue circles represent isolates from water and sediment. **(A)** The tree consists of a 619 bp alignment based on the *polB* partial sequence. **(B)** The tree consists of an 813 bp alignment based on the BTB/POZ domain-containing protein gene partial sequence.

The multibanded pandoravirus strains were purified, and the cut PCR fragments were successfully reamplified using the same primers for subsequent sequencing analysis (Figure 2B, Lanes 3 and 4). Consequently, the *polB* gene region was decoded in five of the six isolates, whereas a portion of the BTB/POZ domain-containing protein was decoded in the remaining isolate. The breakdown of *Pandoravirus* isolates revealed three out of six isolates of Lineage A and three out of six isolates of Lineage B (Figure 5).

**Figure 6:**
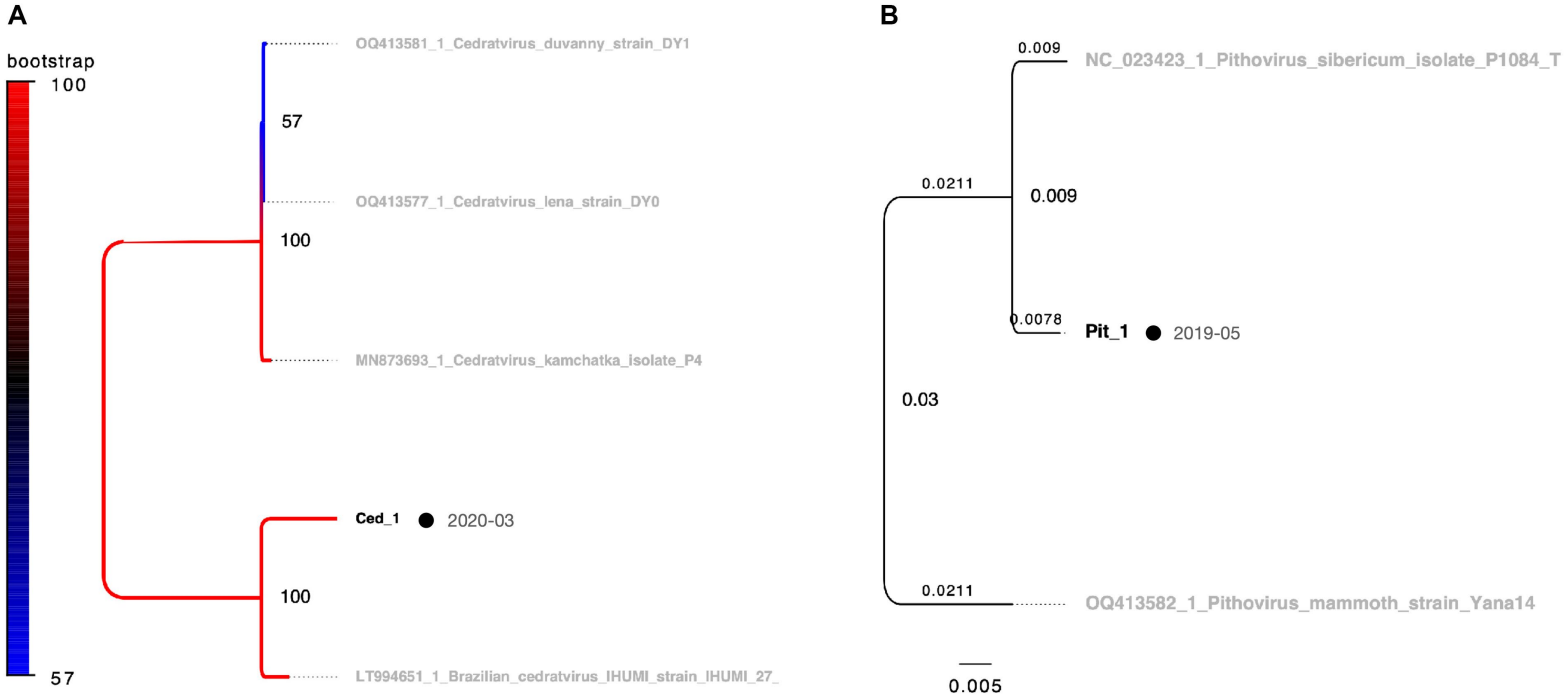
Phylogenetic classification results for *Cedratvirus* and *Pithovirus.* OTUs in black represent isolates in this study, while those in grey denote known strains. Black circles indicate isolates from soil samples, which are consistent with the other figures. The date adjacent to the isolate indicates the month of isolation. **(A)** *Cedratvirus* molecular phylogenetic tree using 703 bp alignment data based on the partial sequence of the ankyrin repeat-containing protein gene. **(B)** *Pithovirus* molecular phylogenetic tree using alignment data of 687 bp based on the partial sequence of the triple helix repeat protein gene. In panel **(B)**, branch labels indicate branch times and node labels indicate node age.

Remarkably, the genomic fragments of the two heterologous viral strains were successfully amplified using the *polB* primers of the pandoravirus (Figures 2B,C). One isolate, identified as *Cedratvirus*, was isolated from the soil in March 2020, showing amplification of a part of the ankyrin repeat-containing protein-coding gene. Phylogenetic analysis revealed its close relationship with the Brazilian Cedratvirus IHUMI (Figure 2B, Lane 7, Figure 6A). The other isolate, *Pithovirus*, exhibited amplification of a portion of the collagen triple helix repeat protein-coding gene and demonstrated a close relationship with *Pithovirus sibericum* P1084 based on phylogenetic relationships (Figures 2C, 6B). Both viruses were isolated from soil samples (Figure 6).

### 3.2 Analysis of month-to-month variation in the number of isolates in giant viruses isolated from brackish soil

**Figure 7:**
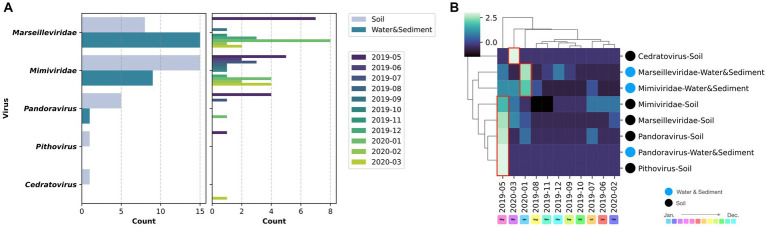
Virus source and temporal variation. **(A)** The left column presents the number of isolates based on sample type for each virus, while the right column illustrates the number of isolates per month; **(B)** Clustering of the matrix of viral isolates per sample type per month.

Comparing the sources of virus isolates over the course of the year, eight isolates of marseilleviruses originated from soil, whereas 15 were obtained from water and sediment. For mimiviruses, 15 isolates were sourced from soil and nine from water and sediment. Five *Pandoravirus* isolates were obtained from soil and one each from water and sediment (Figure 7A). Examination of the molecular phylogenetic data for pandoraviruses revealed that the isolation timing diverged from those of *Marseilleviridae* and *Mimiviridae*, with four of the six isolates obtained in May 2019. The other periods were July 2019 and January 2020, each with one isolate (Figures 5, 7A). Notably, only one of the six pandoravirus strains was isolated from water containing riverbed sediment, highlighting a bias in both timing and source compared with marseilleviruses and mimiviruses (Figure 7A). The top three-monthly isolates for marseilleviruses were eight in January 2020, seven in May 2019, and three in December 2019; for mimiviruses, five in May 2019; and four each in January 2020 and March 2020, showing a skewed distribution in the spring and winter months. Notably, no month throughout the year lacked a single giant virus isolation; however, in August, October, and November 2019, there was only one isolate each (August: *Mimivirus* from water and sediment samples; October: *Cotonvirus* from soil samples; November: Lineage B *Marseillevirus* from water and sediment samples) (Figures 4, 5, 7A).

Cluster mapping of the number of isolates of each virus based on the source and time of isolation categorized *Pandoravirus*, *Pithovirus*, and soil-derived *Marseilleviridae* and *Mimiviridae* as the May 2019-derived sample groups. Water- and sediment-derived *Marseilleviridae* and *Mimiviridae* were classified as the January 2020 group, whereas *Cedratvirus* (soil-derived), isolated in March 2020, formed an independent cluster. In summary, soil-derived viruses, excluding *Cedratvirus*, exhibited a bias toward May 2019, whereas the water sample-derived viruses *Marseilleviridae* and *Mimiviridae* demonstrated a bias toward the January 2020 isolation period (Figure 7B).

When the number of viral isolates from soil and water samples was quantified per gram of soil or per milliliter of water sample, the highest number of isolates from soil samples for a single sample occurred in July 2019, whereas the highest number of isolates from water samples was observed in January 2020. In May 2019, virus isolation was successful in all three soil samples collected each month. However, in the other months, some samples yielded no detectable viruses (Table 1).

**Table 1:** Number of virus isolates per soil and water sample for each sample.

Year-month	Soil (Isolates/g)			Water and sediment (Isolates/mL)
2019-05	9.3	4.9	1.8	2.4
2019-06	2.4	1.8	–	–
2019-07	12.8	–	–	0.4
2019-08	–	–	–	0.4
2019-09	1.2	–	–	0.4
2019-10	1.0	–	–	–
2019-11	–	–	–	0.4
2019-12	1.4	–	–	1.2
2020-01	3.9	–	–	4.0
2020-02	4.1	1.3	–	–
2020-03	5.3	–	–	0.8

## 4 Discussion

In general, the environmental virosphere exhibits considerable diversity, with viruses pervasive in various ecosystems, ranging from aquatic regions to soil environments (Breitbart and Rohwer, 2005; Pratama and van Elsas, 2018). It is imperative to emphasize that the identification of viruses across diverse environments and the spatiotemporal dynamics of their distribution constitute two distinct avenues of research. The former signifies the ubiquity of viruses globally, reaffirming their presence across different geographical locations while the latter delves into the role of giant viruses within ecosystems by probing the intricacies of their emergence, spatial distribution, and temporal dynamics. Giant viruses, akin to their smaller counterparts, display diverse distributions and have been isolated from various locations and sample types including reservoir water, oceans, Siberian permafrost, submarine soil, and hypersaline lakes (Raoult et al., 2004; Arslan et al., 2011; Philippe et al., 2013; Legendre et al., 2014; Abrahão et al., 2018). The present study began with the isolation of two pandoravirus isolates and one mimivirus isolate from soil in April 2019 (Akashi and Takemura, 2019). Subsequently, extensive sampling was conducted at the same site over approximately one year, from May 2019 to March 2020, with the aim of recurrently isolating giant viruses from both soil and water samples. The results of this effort revealed the continuous isolation of giant viruses representing five genera, confirming the ubiquitous presence of giant viruses in the global environment (Figures 2–6).

This study presents a snapshot of the annual isolation of giant viruses from the soil and water within a specific riverbed in Japan. The duration of this observation, limited to one year, precluded the extrapolation of continuous trends. The presence of microorganisms and viruses in the environment, not only giant viruses, depends on whether the environment is habitable, i.e., the environmental context. Therefore, the results of studies similar to this one, on targets in the natural environment are greatly affected by large-scale natural disasters such as tsunamis, earthquakes, and typhoons, as well as human-induced environmental modifications such as construction, and changes in the habitats of organisms involved in virus transmission. We believe that these research limitations can be overcome by conducting continuous and detailed monitoring of the sampling sites in parallel with sampling. Consequently, these findings lack direct applicability to diverse natural environments. Despite this limitation, existing literature suggests a geographic bias in the distribution of giant viruses over several square kilometers (Boratto et al., 2015, 2018; Tan et al., 2018). Additionally, our study revealed an intra-square meter imbalance in the number of viral isolates, further underscoring the spatial disparities involved. The observed equilibrium in the number of viral isolates indicates a distinct spatiotemporal bias in the isolation of giant viruses (Table 1). A noteworthy limitation of this study was the absence of isolated host organisms. Nevertheless, the absence of temporal bias in the isolation of most subphylogenetic clades of *Mimiviridae* and *Marseilleviridae* suggests the potential for the concurrent propagation of multiple phylogenetic clades co-infecting specific hosts (Figures 3, 4). Regarding the proliferation site, it remains uncertain whether the isolated giant viruses proliferated locally or originated from a distant proliferative source. However, the uninterrupted isolation of giant viruses throughout the year indicates their proximity to a region that exhibits substantial proliferation.

Two plausible interpretations arise concerning the relationship between the recurrent isolation of giant viruses from river soils and their potential hosts. The first interpretation posits that both the giant virus and its host were present and actively proliferating near the sampling site. The second interpretation suggests that the giant virus transiently passes through the sampling site. If proliferation occurs on-site, a higher recovery quantity would be expected from the soil, whereas if the virus flows through, a greater number of isolates would be anticipated from water and sediment samples. Examining the distribution of viral isolates by sample type in our study, *Marseilleviridae* were more frequently isolated from water samples with sediment than from soil, indicating a potential influx via river flow. Conversely, *Mimiviridae* and *Pandoravirus* exhibited higher isolation rates from soil, implying a substantial presence of these viruses in the terrestrial environment surrounding the sampling sites. The isolation of *Pithovirus* and *Cedratvirus* from soil, akin to *Pandoravirus*, supports this observation. Although *Cedratvirus* and *Pithovirus* share an ostiole-like structure with *Pandoravirus*, the prevalence of soil-isolated pandoravirus strains (5/6 strains, approximately 80%, Figure 7A) suggests that ostiole-like virus particles might exhibit limited mobility in the environment, and are preferentially isolated from the soil proximal to their proliferative sites, aligning with the first interpretation. The efficiency of virus isolation exhibited temporal variation; however, the precise attribution of this variability to the sample collection point or potential temporal fluctuations in virus abundance remains unresolved, making this a significant avenue for future investigations.

As an illustration of the ecological transmission of viruses, mimivirus particles attach to the legs of mosquitoes (*Aedes* spp.), suggesting that giant viruses can spread through the mobility of insects (Rodrigues et al., 2015). Furthermore, recent reports have shown that noroviruses can be carried by birds (Sato et al., 2024). Given the mobility of these organisms, it is conceivable that the virus can spread from nearby locations of giant viral proliferation. This underscores the need for comprehensive research to elucidate the ecological transmission pathways of giant viruses in brackish soils.

## 5 Conclusion

This study contributes to the understanding of the ubiquitous presence of giant viruses in the environment by investigating the reproducibility of their isolation. These findings indicate a non-uniform distribution of giant viruses within an area of a few square meters, emphasizing the existence of localized hotspots in the environment with a heightened probability of giant virus isolation.

## Data availability statement

The original contributions of this study are included in the article/Supplementary material. Further inquiries may be directed at the corresponding author.

## Author contributions

MA: Writing – original draft, Writing – review & editing. MT: Writing – review & editing. SS: Writing – review & editing.

## Footnotes

^1^ https://www.jma.go.jp/jma/index.html

## Supplementary material

SUPPLEMENTARY FILE S1 PCR cycling parameters/DNA sequences of decoded virus isolates.

SUPPLEMENTARY FILE S2 OTU list of known viruses used during molecular phylogenetic analysis.
