# Supplementary material for: Continuous year-round isolation of giant viruses from brackish shoreline soils
Source: Front Microbiol. 2024 May 2;15:1402690. doi: 10.3389/fmicb.2024.1402690 (PMC11096492; doi:10.3389/fmicb.2024.1402690)
Supplement: SUPPLEMENTARY FILE S1 — PCR cycling parameters/DNA sequences of decoded virus isolates. [file Data_Sheet_1.pdf]

Supplementary Material

Continuous Year-Round Isolation of Giant Viruses from Brackish Shoreline Soils

Motohiro Akashi[1*], Masaharu Takemura[2], Seiichi Suzuki[1]

[1]Department of Science and Technology, Faculty of Science and Technology, Seikei University, 3-3-1 Kichijojikitamachi, Musashino-shi, Tokyo 180-8633, Japan

[2]Institute of Arts and Sciences, Tokyo University of Science, 1-3 Kagurazaka, Shinjuku-ku, Tokyo 162-8601, Japan

*** Correspondence:**
Motohiro Akashi
motohiro-akashi@st.seikei.ac.jp

1 PCR Cycling Parameters

1.1 *Mimivirus* and *Marseillevirus*

Step	Temperature	Time	Cycle
Denaturation	94 ˚C	1 min	1 cycle
Denaturation	98 ˚C	10 sec	35 cycles
Annealing	55 ˚C	15 sec	
Extension	68 ˚C	1 min	
Extension	68 ˚C	7 min	1 cycle
End/Hold	8 ˚C	To hold	

1.2 *Pandoravirus*

Step	Temperature	Time	Cycle
Denaturation	94 ˚C	1 min	1 cycle
Denaturation	98 ˚C	10 sec	5 cycles
Annealing	55 ˚C	15 sec	
Extension	68 ˚C	1 min	
Denaturation	98 ˚C	10 sec	35 cycles
Annealing	60 ˚C	15 sec	
Extension	68 ˚C	1 min	
Extension	68 ˚C	7 min	1 cycle
End/Hold	8 ˚C	To hold	

1.3 *Pithovirus*

Step	Temperature	Time	Cycle
Denaturation	94 ˚C	1 min	1 cycle
Denaturation	98 ˚C	10 sec	
Annealing	24.3 ˚C	15 sec	40 cycles
Extension	68 ˚C	1 min	
End/Hold	8 ˚C	To hold	1 cycle

1.4 *Cedratvirus*

Step	Temperature	Time	Cycle
Denaturation	94 ˚C	1 min	1 cycle
Denaturation	98 ˚C	10 sec	
Annealing	11.6 ˚C	15 sec	40 cycles
Extension	68 ˚C	1 min	
End/Hold	8 ˚C	To hold	1 cycle

2 DNA Sequences of Decoded Virus Isolates

>Mal_1

TGTTGGTCACACCAGCAGAGGCGTTGACGGCAGCGGTCGAAGGAACGTACTGGTTCGACACGTTGGT
GAGCTTGGAATAATTGGTCGAACCCTTGGGGTCAGAGGCGAAAGTCTCCAGCGAGTACGAGTACGCG
TGGTAGCCGGTCTCCTCGGGGATCGACTTGTGCCAGTACCACGGCACAACAAGGCTGTAATAGTCGG
AACCGTTGCTGACACGGGCAGTCGACTCGTACACAAGCTGGGCAGCCTCAAGCGGGTCAAGACCGGC
ATAGGCGGGCTCGGTGGTGTAGTTGGACCATTCGCCGGGAGTGGAGCTGTTTCGGATGGCGTAGAAG
TATCCAACAACGGCGTGAGAGATACGGATATCAATCGGGATGAGAGCGTTGAGCTGCGAAAGGTTGA
TGGTCGTCTCATTGACCTTCTGAACCTGCTTGATAACCATATCACGGGGATTCTTACCCATCTTGAC
ACGTTCGTCATTGTGCACGACGGCGTAATGGCACCAGGTTTCGACATTGGTGATGGCGGGAGGGTTG
GAGCTGTAAATGAGGGTGAAAGTGGCATCGTAGCTCACCTGAACAACATCGTCGAGAGTGGGAGGGT
TGGCGCCAACACCCACGTTCAGGACGAGAAGATCCTGCCACCTGCGGAGACAGTAGTTAATCTTGAT
GTCGTTGAAGGGAAGGGCCGAAACGGCAAGGGCCAGACCCGAGTCCTCCGAATAGAAAAGGGGGATA
GGAAGGTTGAAGAATTCGCCGGTGCCGAGGGGGTTACCGGTCGTCACAGGGTTAATCATGGCGGGAA
TGTCTCCAATCATGTTCCTGTAACCGACACGCTTGGAAGCATCAATATTGAACTGAGAGTTAAAGTC
GAACCAATAGCTGTCAAACTCATGAACAATCAGATCGTTGAAAGTGATGTTAATCTTTTCAACAAGA
TTGTGCATAAAGTTCCTGGTCCAGCGGATGGTGGCATTAGCATTGATGGCGCCACCAGCGTTGGTCG
GGCGAATGGCAATCAGAGGGAGACGGACACGCAGCCAGGTGTTGAGGACGTAATCACCGGAGCGATT
CACAGACGCGGAGAATTCATTACCGAAGCCGGGAAGACCCGAAATATTTCGGAGAACAACGGGAAGG
AAGGAGAACCAATTGGCCTTTTTGATGGCACGCACAAAATAGGTGACTGCCGAGCAACCACCATAGA
GATACGCCTCCAAGTCAGAAAA

>Mal_2

ACCTTCTCTGGACTTGGAGGCGTATCTTTACGGTGGTTGCTCGGCAGTCACCTATTTCGTGCGTGCC
ATCAAAAAGGCCAATTGGTTCTCCTTCCTTCCCGTTGTTCTCCGAAATATTTCGGGTCTTCCCGGAT
TCGGTAATGAATTCTCCGCGTCTGTGAATCGCTCCGGTGATTACGTCCTCAACACCTGGCTGCGTGT
CCGTCTCCCTCTGATTGCCATTCGCCCGACCAACGCTGGTGGCGCCATCAATGCTAATGCCACCATC
CGCTGGACCAGGAACTTTATGCACAATCTTGTTGAAAAGATTAACATCACTTTCAACGATCTGATTG
TTCACGAGTTTGACAGCTATTGGTTCGACTTTAACTCTCAGTTCAATATTGATGCTTCCAAGCGCGT
CGGTTACAGGAACATGATTGGAGACATCCCCGCCATGATCAACCCTGTGACGACCGGTAACCCCCTC
GGCACCGGCGAATTCTTCAACCTTCCTATCCCCCTCTTCTATTCAGAGGACTCAGGTCTGGCTCTTG
CCGTTTCGGCCCTTCCTTTCAACGACATCAAGATTAACTACTGTCTCCGCAGGTGGCAGGATCTTCT
CGTCCTGAACGTGGGTGTTGGCGCCAACCCTCCCACTCTCGACGATGTTGTTCAGGTGAGCTACGAT
GCCACTTTCACCCTCATTTACAGCTCCAACCCTCCCGCCATCACCAATGTCGAGACTTGGTGCCACT
ATGCCGTCGTGCACAATGACGAACGTGTCAAGATGGGTAAGAATCCCCGTGATATGGTTATCAAGCA
GGTTCAGAAGGTCAATGAGACGACCATCAACCTTTCGCAGCTCAACGCTCTCATCCCGATTGATATC
CGTATCTCTCACGCCGTTGTTGGCTACTTCTACGCCATCCGAAACAGCTCCACTCCCGGCGAATGGT
CCAACTACACCACCGAGCCCGCCTATGCCGGTCTTGACCCGCTTGAGGCTGCCCAGCTCGTGTACGA
GTCGACTGCCCGTGTCAGCAACGGTTCCGACTATTACAGCCTTGTTGTGCCGTGGTACTGGCACAAG
TCGATCCCCGAGGAGACCGGCTACCACGCGTATTCGTACTCGCTGGAGACTTTCGCTTCTGACCCCA
AGGGTTCGACCAATTATTCCAAGCTCACCAACGTGTCGAACCAGTACGTTCCTTCGACCGCTGCCGT
CAACGCCTCTGCTGGCGTGACCAACACCGGTATT

>Mal_3

CGACCTTCTCTGACTTGGAGGCGTATCTTTACGGTGGTTGCTCGGCAGTCACCTATTTCGTGCGTGC
CATCAAAAAGGCCAATTGGTTCTCCTTCCTTCCCGTTGTTCTCCGAAATATTTCGGGTCTTCCCGGA
TTCGGTAATGAATTCTCCGCGTCTGTGAATCGCTCCGGTGATTACGTCCTCAACACCTGGCTGCGTG
TCCGTCTCCCTCTGATTGCCATTCGCCCGACCAACGCTGGTGGCGCCATCAATGCTAATGCCACCAT
CCGCTGGACCAGGAACTTTATGCACAATCTTGTTGAAAAGATTAACATCACTTTCAACGATCTGATT
GTTCACGAGTTTGACAGCTATTGGTTCGACTTTAACTCTCAGTTCAATATTGATGCTTCCAAGCGCG
TCGGTTACAGGAACATGATTGGAGACATCCCCGCCATGATCAACCCTGTGACGACCGGTAACCCCCT
CGGCACCGGCGAATTCTTCAACCTTCCTATCCCCCTCTTCTATTCAGAGGACTCAGGTCTGGCTCTT
GCCGTTTCGGCCCTTCCTTTCAACGACATCAAGATTAACTACTGTCTCCGCAGGTGGCAGGATCTTC
TCGTCCTGAACGTGGGTGTTGGCGCCAACCCTCCCACTCTCGACGATGTTGTTCAGGTGAGCTACGA
TGCCACTTTCACCCTCATTTACAGCTCCAACCCTCCCGCCATCACCAATGTCGAGACTTGGTGCCAC
TATGCCGTCGTGCACAATGACGAACGTGTCAAGATGGGTAAGAATCCCCGTGATATGGTTATCAAGC
AGGTTCAGAAGGTCAATGAGACGACCATCAACCTTTCGCAGCTCAACGCTCTCATCCCGATTGATAT
CCGTATCTCTCACGCCGTTGTTGGCTACTTCTACGCCATCCGAAACAGCTCCACTCCCGGCGAATGG
TCCAACTACACCACCGAGCCCGCCTATGCCGGTCTTGACCCGCTTGAGGCTGCCCAGCTCGTGTACG
AGTCGACTGCCCGTGTCAGCAACGGTTCCGACTATTACAGCCTTGTTGTGCCGTGGTACTGGCACAA
GTCGATCCCCGAGGAGACCGGCTACCACGCGTATTCGTACTCGCTGGAGACTTTCGCTTCTGACCCC
AAGGGTTCGACCAATTATTCCAAGCTCACCAACGTGTCGAACCAGTACGTTCCTTCGACCGCTGCCG
TCAACGCCTCTGCTGGCGTGACCAACACCGGTATTCC

>Mal_4

GGCGACCTTCTCCGACTTGGAGGCGTACCTTTATGGTGGTTGCTCGGCGGTCACCTATTTCGTGCGT
GCCATCAAAAAGGCTAATTGGTTCTCTTTCCTCCCCGTTGTTCTCCGAAACATTTCGGGCCTTCCCG
GCTTTGGTAACGAATTCTCCGCGTCTGTGAATCGTTCCGGCGATTACGTCCTCAACACCTGGCTTCG
CGTCCGTCTTCCTCTCATTGCTATCCGTCCGACCAATGCTGGTGGTGCCATTAACGCTAACGCCACC
ATCCGCTGGACCCGAAACTTCATGCACAATCTTGTTGAAAAGATCAATATCACCTTCAATGATCTGA
TTGTTCACGAGTTTGACAGCTACTGGTTCGACTTTAACTCCCAGTTCAACATCGATGCTTCCAAGCG
CGTCGGTTACAGGAACATGATCGGAGACATTCCCGCCATGATCAACCCCGTGACGACCGGCAACCCC
CTCGGCACCGGCGAATTCTTCAATCTTCCCATCCCCCTCTTCTATTCCGAGGACTCGGGTCTGGCTC
TTGCCGTCTCTGCCCTTCCTTTCAACGACATCAAGATCAACTACTGTCTCCGCAGGTGGCAGGATCT
TCTTGTCCTGAATGTGGGTGTTGGCGCCAACCCTCCCACTTTCGACGATGTTGTTCAGGTGAGCTAC
GACGCCACCTTCACCCTCATTTACAGCTCGAACGCTCCCGCCATCACCAATGTCGAGACTTGGTGCC
ACTACGCCGTTGTGCACAATGACGAACGTGTCAAGATGGGTAAGAATCCCCGTGATATGGTCATCAA
GCAGGTTCAAAAGGTCAATGAGACGACCATCAATCTTTCGCAGCTCAACGCTCTCATCCCGATTGAT
ATCCGTATCTCTCACGCCGTCGTTGGCTATTTCTACGCCATCAGGAACAGCTCCACTCCCGGCGAAT
GGTCCAACTACACCACCGAGCCCGCCTATGCCGGTCTTGACCCGCTTGAGGCTGCCCAGCTTGTGTA
CGAGTCGACTGCCCGTGTCAGCAACGGTTCCGACTATTACAGCCTTGTTGTGCCGTGGTACTGGCAC
AAGTCGATCCCCGAGGAGACCGGCTACCACGCGTACTCGTACTCTCTGGAGACTTTCGCCTCTGATC
CCAAGGGTTCGACCAATTATTCCAAGCTCACCAACGTGTCGAACCAGTACGTTCCTTCGACCGCTGC
CGTCAACGCCTCTGCTGGTGTGACCAACACCGGTATTCCGATT

>Mal_5

TTTCTCGGATTTGGAGGCTTACCTCTATGGTGGCTGTTCCGCCGTCACCTATTTTGTGCGTGCCATC
AAAAAGGCCAATTGGTTCTCCTTCCTCCCTGTTGTTCTCCGTAACATCTCGGGTCTTCCCGGCTTCG
GTTCAGAGTTCTCTGCTTCTGTGAATCGTTCCGGAGATTACGTCCTCAACACCTGGCTGCGTGTGCG
TCTTCCTCTCGTGGCCATTCGTCCCACCAACACTGGTGGCGCCATCAACGCTAACGCCACCATTCGC

TGGACCAGAAACTTCATGCACAATCTTGTGGAGAAGGTTAACATCACTTTCAATGACCTCATCGTCC
ATGAGTTTGACAGCTACTGGTTCGACTTCAACTCGCAGTTCAACATCGACGCTTCCAAGCGCGTCGG
TTACAGGAACATGATCGGAGATATTCCGGCCATGATTAACCCTGTGACGACCGGCAACCCTCTCGGC
ACTGGCGAGTTCTTCAATCTTCCCATTCCTCTCTTCTACACCGAGGATTCCGGTCTCGCCCTTGCTG
TGTCGGCTCTTCCGTTCAACGACATCAAGATTAACTACTGTCTTCGCAGGTGGCAGGATCTGATTGT
CCTCAACGTGGGCGTCGGCGGTAACCCTCCCACTTATGACGACATTGTCCAGGTTTCTTACGACTCT
ACTTTCACCCTCATCTACAGTTCGAACGCTCCCGCCATCACCAATGTCGAGACCTGGTGTCACTACG
CCGTCGTCCACAACGACGAACGCGTCAAGATGGGTAAGAATCCCCGTGACATGGTCATCAAGCAGGT
GCAAAAGGTCAACGAGACGACCATCAACCTTTCACAGCTCAACGCCCTTGTCCCCATCGACATTCGC
GTGTCTCATGCCGTCGTTGGATATTTCTACGCTATTCGAAACAGTTCGACCACTGGTGAATGGTCCA
ATTACACCACTGAGCCTGCCTATGCTGGTCTCGACCCTCTTGAGGCCGCTCAGCTCGTATACGAGTC
GACTGCCCGTGTCAGCAACGGTTCTGACTATTACAGCCTGATGGTGCCGTGGTACTGGCACAAGTCG
ATCCCCGAGGAGACGGGTTACCACGCGTACTCTTATTCTCTCGACACCTTTGCTTCTGACCCCAAGG
GTTCGACCAATTATTCCAAGCTCACTAACGTGTCGAACCAGTACGTTCCTTCGACCGCTGCTGTCAA
CGCTTCTGCTGGCGTGACTAACACCGGCATTCCGATCCCTCGG

>Mal_6

GGCGAACCTTCTCTGACTTGGAGGCGTATCTTTACGGTGGTTGCTCGGCAGTCACCTATTTCGTGCG
TGCCATCAAAAAGGCCAATTGGTTCTCCTTCCTTCCCGTTGTTCTCCGAAATATTTCGGGTCTTCCC
GGCTTCGGTAATGAATTCTCCGCGTCTGTGAATCGCTCCGGTGATTACGTCCTCAACACCTGGCTGC
GTGTCCGTCTCCCTCTGATTGCCATTCGCCCGACCAACGCTGGTGGCGCCATCAATGCCAATGCTAC
CATTCGCTGGACCAGGAACTTTATGCACAATCTTGTTGAAAAGGTTAACATCACTTTCAACGATCTG
ATTGTTCATGAGTTTGACAGCTATTGGTTCGACTTTAACTCTCAGTTCAACATTGATGCTTCCAAGC
GGGTCGGTTACAGGAACATGATTGGAGACATCCCCGCCATGATCAACCCTGTGACGACTGGTAACCC
CCTCGGCACCGGCGAATTCTTTAATCTTCCCATTCCCCTCTTTTATTCAGAGGACTCGGGTCTGGCT
CTTGCCGTTTCAGCCCTTCCCTTCAACGACATCAAGATTAACTACTGTCTCCGCAGGTGGCAGGATC
TTCTTGTTCTGAACGTGGGTGTTGGCGCCAACCCTCCCACTCTCGACGATGTTGTTCAGGTGAGCTA
CGATGCCGCCTTCGCCCTCGTCTACAGCTCGAACCCTCCCGCCATCACCAATGTCGAGACCTGGTGC
CATTATGCCGTTGTTCACAATGACGAACGTGTCAAGATGGGTAAGAATCCCCGTGATATGGTCATCA
AGCAGGTCCAGAAGGTCAACGAGACGACCATCAACCTTTCGCAGCTCAACGCTCTCATCCCGATTGA
CATCCGTATCTCTCACGCCGTTGTTGGCTACTTCTACGCCATCCGAAACAGCTCCACTCCCGGCGAA
TGGTCCAACTACACCACCGAGCCCGCCTATGCCGGTCTTGACCCGCTTGAGGCTGCCCAGCTTGTAT
ACGAGTCGACCGCCCGTGTCAGCAACGGTTCTGACTATTACAGTCTTGTTGTGCCGTGGTACTGGCA
CAAGTCGATCCCCGAGGAAACCGGCTACCACGCGTATTCGTACTCGCTGGAGACTTTCGCCTCTGAC
CCCAAGGGTTCGACCAATTATTCCAAGCTCACCAACGTGTCGAACCAGTACGTTCCTTCGACCGCTG
CCGTCAACGCCTCTGCTGGCGTGACCAACACCGGTATTCC

>Mal_7

GACTTTCTCCGATTTGGAGGCCTACCTCTATGGTGGTTGTTCTGCCGTCACTTATTTTGTGCGTGCC
ATCAAAAAGGCTAATTGGTTCTCTTTCCTCCCCGTCGTTCTCCGCAACATCTCGGGTCTTCCCGGTT
TCGGCTCAGAGTTTTCCGCCTCTGTGAATCGTTCTGGCGATTACGTCCTCAACACTTGGCTGCGCGT
GCGTCTGCCCCTCATCGCCATCCGCCCGACCAATGCTGGTGGCGCCATCAACGCTAACGCCACTATC
CGATGGACCAGGAACTTCATGCACAACCTTGTCGAGAAGGTCAACATCACTTTCAATGACCTCATCG
TCCACGAGTTTGACAGCTATTGGTTTGACTTCAACTCCCAGTTCAACATCGACGCTTCCAAGCGCGT
CGGTTACAGGAACATGATTGGAGATATTCCCGCCATGATTAATCCCGTGACGACTGGCAACCCCCTC
GGAACCGGTGAGTTCTTCAATCTTCCCATTCCTCTCTTCTACACCGAAGATTCCGGTCTCGCTCTTG
CTGTGTCTGCTCTTCCTTTCAACGACATCAAGATCAACTACTGCCTTCGCAGGTGGCAAGACCTGAT

TGTCCTCAACGTGGGTGTCGGTGCCAATCCTCCCACGTTTGACGACATTGTCCAGGTTTCTTACGAT
GCCGCTTTCACTCTCATCTACAGCTCAAACGCTCCCGCCATCACCAACGTCGAGACTTGGTGCCACT
ACGCCGTTGTGCACAATGACGAGCGTGTCAAGATGGGTAAGAATCCCCGTGATATGGTCATCAAGCA
GGTGCAAAAGGTCAACGAGACGACCATCAACCTTTCGCAGCTCAACGCTCTTGTTCCCATCGACATC
CGCGTGTCGCATGCCGTTGTCGGTTACTTCTACGCTATCAGGAACAGCTCCACCCCTGGCGAATGGT
CCAATTACACCACCGAGCCCGCCTATGCTGGTCTTGACCCGCTCGAGGCTGCCCAGCTTGTGTACGA
GTCTACCGCCCGTGTCAGCAACGGTTCTGATTATTACAGCCTCATGGTGCCGTGGTTCTGGCACAAA
TCCATCCCGGAAGAGACCGGCTACCACGCGTACTCGTACTCTCTCGACACGTTCGCTTCTGACCCCA
AGGGTTCGACCAATTATTCCAAGCTCACCAATGTGTCGAACCAGTATGTCCCTTCAGTCGCTGCTGT
CAACGCCTCTGCTGGTGTGACCAACACCGG

>Mal_8

GGCGACCTTCTCCGACTTGGAGGCGTATCTCTATGGTGGTTGCTCGGCAGTCACCTATTTCGTGCGT
GCCATCAAAAAGGCCAATTGGTTCTCCTTCCTTCCCGTTGTTCTCCGAAATATTTCGGGTCTTCCCG
GCTTCGGTAATGAATTCTCCGCGTCTGTGAATCGCTCCGGTGATTACGTCCTCAACACCTGGCTGCG
CGTCCGTCTCCCTCTGATTGCTATTCGCCCGACCAATGCTGGTGGCGCCATCAATGCTAACGCCACC
ATCCGCTGGACCAGGAACTTTATGCACAATCTTGTTGAAAAGGTCAATATCACCTTCAATGATCTGA
TTGTTCACGAGTTTGACAGCTACTGGTTCGACTTTAACTCTCAGTTCAATATTGATGCTTCCAAGCG
CGTCGGTTACAGGAACATGATTGGAGACATTCCCGCCATGATTAACCCTGTGACGACCGGCAACCCC
CTCGGCACCGGCGAATTCTTCAACCTTCCTATCCCCCTCTTTTATTCAGAGGACTCGGGTCTGGCCC
TTGCCGTTTCAGCCCTTCCCTTCAACGACATCAAGATTAACTACTGTCTCCGCAGGTGGCAGGATCT
TCTCGTCCTGAACGTGGGTGTTGGCGCCAACCCTCCCACTCTCGACGATGTTGTTCAGGTGAGCTAC
GATGCCACCTTCACCCTGATTTACAGCTCCAACCCTCCCGCCATCACCAATGTCGAGACTTGGTGCC
ACTACGCCGTCGTGCACAATGACGAGCGTGTCAAGATGGGTAAGAATCCCCGTGATATGGTCATCAA
GCAGGTTCAGAAGGTCAATGAGACGACCATCAACCTTTCGCAGCTCAACGCTCTCATCCCGATTGAT
ATCCGTATCTCTCACGCCGTTGTTGGATACTTCTACGCCATCCGAAACAGCTCCACTCCCGGCGAAT
GGTCCAACTACACCACCGAGCCCGCCTATGCCGGTCTTGACCCGCTTGAGGCTGCCCAGCTTGTTTA
CGAGTCGACTGCCCGTGTCAGCAACGGTTCCGACTATTACAGTCTTGTTGTGCCGTGGTACTGGCAC
AAGTCGATCCCCGAGGAGACCGGCTACCACGCGTACTCGTACTCGCTGGAGACTTTCGCCTCTGACC
CCAAGGGTTCGACCAATTATTCCAAGCTCACCAATGTGTCGAACCAGTACGTTCCTTCGACTTCTGC
CGTCAACGCCTCTGCTGGTGTGACCAACACCGG

>Mal_9

GGCGACCTTCTCTGACTTGGAGGCGTATCTCTATGGTGGTTGCTCGGCAGTCACCTATTTTGTGCGT
GCCATCAAAAAGGCCAATTGGTTCTCCTTCCTTCCCGTTGTTCTCCGAAATATTTCGGGTCTTCCCG
GCTTCGGTAATGAATTCTCCGCGTCTGTGAATCGCTCCGGTGATTACGTCCTCAACACCTGGCTGCG
CGTCCGTCTCCCTCTGATTGCTATTCGCCCGACCAACGCTGGTGGCGCCATCAATGCTAACGCCACC
ATCCGCTGGACCAGGAACTTTATGCACAATCTTGTTGAAAAGATTAACATCACTTTCAACGATCTGA
TTGTTCATGAGTTTGACAGCTATTGGTTCGACTTTAACTCTCAGTTCAATATTGATGCTTCCAAGCG
TGTCGGTTACAGGAACATGATTGGAGACATTCCCGCCATGATTAACCCTGTGACGACCGGCAACCCC
CTCGGCACCGGCGAATTCTTCAACCTTCCTATCCCCCTCTTTTATTCAGAGGACTCGGGTCTGGCCC
TTGCCGTTTCGGCCCTTCCCTTCAACGACATCAAGATTAACTACTGTCTCCGCAGGTGGCAGGATCT
TCTCGTCCTGAACGTGGGTGTTGGCGCCAACCCTCCCACTCTCGACGATGTTGTTCAGGTGAGCTAC
GATGCCACTTTCACCCTCATTTACAGCTCCAACCCTCCCGCCATCACCAATGTCGAAACCTGGTGCC
ATTACGCCGTCGTGCACAATGACGAACGTGTCAAGATGGGTAAGAATCCCCGTGATATGGTTATCAA
GCAGGTTCAGAAGGTCAATGAGACGACCATCAACCTTTCGCAGCTCAACGCTCTCATCCCGATTGAT

ATCCGTATCTCTCACGCCGTTGTTGGATACTTCTACGCCATCCGAAACAGCTCCACTCCCGGCGAAT
GGTCCAACTACACCACCGAGCCCGCCTATGCCGGTCTTGACCCGCTTGAGGCTGCCCAGCTTGTGTA
CGAGTCGACTGCCCGTGTCAGCAACGGTTCCGACTATTACAGCCTTGTTGTGCCGTGGTACTGGCAC
AAGTCGATCCCCGAGGAGACCGGCTACCACGCGTACTCGTACTCGCTGGAGACTTTCGCCTCTGACC
CCAAGGGTTCGACCAATTATTCCAAGCTCACCAACGTGTCGAACCAGTACGTTCCTTCGACCGCTGC
CGTCAACGCCTCTGCTGGTGTGACCAACACCGGTATTC

>Mal_10

GGCGACCTTCTCCGACTTGGAGGCGTACCTTTATGGTGGTTGCTCGGCGGTCACCTATTTCGTGCGT
GCCATCAAAAAGGCTAATTGGTTCTCTTTCCTCCCCGTTGTTCTCCGAAACATTTCGGGTCTTCCCG
GTTTCGGTAACGAATTCTCCGCGTCCGTGAATCGTTCCGGCGATTACGTCCTCAACACCTGGCTTCG
CGTCCGTCTTCCTCTCATTGCTATCCGTCCGACCAATGCTGGTGGTGCCATCAACGCTAATGCCACC
ATCCGCTGGACCCGAAACTTCATGCACAATCTTGTTGAAAAGATCAACATCACCTTCAATGATCTGA
TTGTCCACGAGTTTGACAGCTACTGGTTCGACTTTAACTCCCAGTTCAACATCGATGCTTCCAAGCG
CGTCGGTTACAGGAACATGATCGGAGACATTCCCGCCATGATTAACCCCGTGACGACCGGCAACCCC
CTCGGCACCGGCGAATTCTTCAATCTTCCCATCCCCCTCTTCTATTCCGAGGACTCGGGTCTGGCTC
TTGCCGTCTCTGCCCTTCCTTTCAACGACATCAAGATCAACTACTGTCTCCGCAGGTGGCAGGATCT
TCTCGTCCTGAACGTGGGTGTTGGCGCCAACCCTCCCACTTTCGACGATGTTGTTCAGGTGAGCTAC
GACGCCACCTTCACCCTCATTTACAGCTCGAACGCTCCCGCCATCACCAATGTCGAGACTTGGTGCC
ACTACGCCGTTGTGCACAATGACGAGCGTGTCAAGATGGGTAAGAATCCCCGTGATATGGTCATCAA
GCAGGTTCAAAAGGTCAATGAGACGACCATCAATCTTTCGCAGCTCAACGCTCTCATCCCGATTGAT
ATCCGTATCTCTCACGCCGTCGTTGGCTATTTCTACGCCATCAGGAACAGCTCCACTCCCGGCGAAT
GGTCCAACTACACCACCGAGCCCGCCTATGCCGGTCTTGACCCGCTTGAGGCTGCCCAGCTTGTTTA
CGAGTCGACTGCCCGTGTCAGCAATGGCTCTGATTATTACAGCCTTGTTGTGCCGTGGTACTGGCAC
AAGTCGATCCCCGAGGAGACCGGCTACCACGCGTACTCGTACTCTCTGGAGACTTTCGCCTCTGATC
CCAAGGGTTCGACCAATTATTCCAAGCTCACCAATGTGTCGAACCAGTACGTTCCTTCGACCGCTGC
CTTCAACGCCTCTGCTGGTGTGACCAACACCGGTATTCC

>Mal_11

GGCGACCTTCTCTGACTTGGAGGCGTATCTCTATGGTGGTTGCTCGGCAGTCACCTATTTTGTGCGT
GCCATCAAAAAGGCCAATTGGTTCTCCTTCCTTCCCGTTGTTCTCCGAAATATTTCGGGTCTTCCCG
GCTTCGGTAATGAATTCTCCGCGTCTGTGAATCGCTCCGGTGATTACGTCCTCAACACCTGGCTGCG
CGTCCGTCTCCCTCTGATTGCTATTCGCCCGACCAACGCTGGTGGCGCCATCAATGCTAACGCCACC
ATCCGCTGGACCAGGAACTTTATGCACAATCTTGTTGAAAAGATTAACATCACTTTCAACGATCTGA
TTGTTCATGAGTTTGACAGCTATTGGTTCGACTTTAACTCTCAGTTCAATATTGATGCTTCCAAGCG
TGTCGGTTACAGGAACATGATTGGAGACATTCCCGCCATGATTAACCCTGTGACGACCGGCAACCCC
CTCGGCACCGGCGAATTCTTCAACCTTCCTATCCCCCTCTTTTATTCAGAGGACTCGGGTCTGGCCC
TTGCCGTTTCGGCCCTTCCCTTCAACGACATCAAGATTAACTACTGTCTCCGCAGGTGGCAGGATCT
TCTCGTCCTGAACGTGGGTGTTGGCGCCAACCCTCCCACTCTCGACGATGTTGTTCAGGTGAGCTAC
GATGCCACTTTCACCCTCATTTACAGCTCCAACCCTCCCGCCATCACCAATGTCGAGACTTGGTGCC
ATTACGCCGTCGTGCACAATGACGAACGTGTCAAGATGGGTAAGAATCCCCGTGATATGGTTATCAA
GCAGGTTCAGAAGGTCAATGAGACGACCATCAACCTTTCGCAGCTCAACGCTCTCATCCCGATTGAT
ATCCGTATCTCTCACGCCGTTGTTGGATACTTCTACGCCATCCGAAACAGCTCCACTCCCGGCGAAT
GGTCCAACTACACCACCGAGCCCGCCTATGCCGGTCTTGACCCGCTTGAGGCTGCCCAGCTTGTGTA
TGAGTCGACTGCCCGTGTCAGCAACGGTTCCGACTATTACAGCCTTGTTGTGCCGTGGTACTGGCAC
AAGTCGATCCCCGAGGAGACCGGCTACCACGCGTATTCGTACTCGCTGGAGACTTTCGCCTCTGACC

CCAAGGGTTCGACCAATTATTCCAAGCTCACCAACGTGTCGAACCAGTACGTTCCTTCGACCGCCGC
CTTCAACGCCTCTGCTGGTGTGACCAACACC

>Mal_12

TCCGACTTGGAGGCGTACCTTTATGGTGGTTGCTCGGCGGTCACCTATTTCGTGCGTGCCATCAAAA
AGGCTAATTGGTTCTCTTTCCTCCCCGTTGTTCTCCGAAACATTTCGGGCCTTCCCGGCTTTGGTAA
CGAATTCTCCGCGTCTGTGAATCGTTCCGGCGATTACGTCCTCAACACCTGGCTTCGCGTCCGTCTT
CCTCTCATTGCTATCCGTCCGACCAATGCTGGTGGTGCCATTAACGCTAACGCCACCATCCGCTGGA
CCCGAAACTTCATGCACAATCTTGTTGAAAAGATCAATATCACCTTCAATGATCTGATTGTTCACGA
GTTTGACAGCTACTGGTTCGACTTTAACTCCCAGTTCAACATCGATGCTTCCAAGCGCGTCGGTTAC
AGGAACATGATCGGAGACATTCCCGCCATGATCAACCCCGTGACGACCGGCAACCCCCTCGGCACCG
GCGAATTCTTCAATCTTCCCATCCCCCTCTTCTATTCCGAGGACTCGGGTCTGGCTCTTGCCGTCTC
TGCCCTTCCTTTCAACGACATCAAGATCAACTACTGTCTCCGCAGGTGGCAGGATCTTCTCGTCCTG
AACGTGGGTGTTGGCGCCAACCCTCCCACTTTCGACGATGTTGTTCAGGTGAGCTACGACGCCACCT
TCACCCTCATTTACAGCTCGAACGCTCCCGCCATCACCAATGTCGAGACTTGGTGCCACTATGCCGT
TGTGCACAATGACGAGCGTGTCAAGATGGGTAAGAACCCCCGTGATATGGTTATTAAGCAGGTTCAA
AAGGTCAATGAGACGACCATCAACCTTTCGCAGCTCAACGCTCTCATCCCGATTGATATCCGTATCT
CTCACGCCGTCGTTGGCTATTTCTACGCCATTAGGAACAGCTCCACTCCCGGCGAATGGTCCAACTA
CACCACCGAGCCCGCCTACGCCGGTCTTGACCCGCTTGAGGCTGCCCAGCTTGTGTACGAGTCGACT
GCCCGTGTCAGCAATGGCTCTGATTATTACAGCCTTGTTGTGCCGTGGTACTGGCACAAGTCGATCC
CCGAGGAGACCGGCTACCACGCGTACTCGTACTCTCTGGAGACTTTCGCCTCTGATCCCAAGGGTTC
GACCAATTATTCCAAGCTCACCAACGTGTCGAACCAGTACGTTCCTTCGACCGCTGCCGTCAACGCC
TCTGCTGGTGTGACCAACACCGGTATT

>Mal_13

GGCGACCTTCTCTGACTTGGAGGCGTATCTCTATGGTGGTTGCTCGGCAGTCACCTATTTTGTGCGT
GCCATCAAAAAGGCCAATTGGTTCTCCTTCCTTCCCGTTGTTCTCCGAAATATTTCGGGTCTTCCCG
GCTTCGGTAATGAATTCTCCGCGTCTGTGAATCGCTCCGGTGATTACGTCCTCAACACCTGGCTGCG
CGTCCGTCTCCCTCTGATTGCTATTCGCCCGACTAATGCTGGTGGTGCCATTAACGCCAACGCCACC
ATTCGCTGGACCAGGAACTTTATGCACAATCTTGTTGAAAAGATTAACATCACTTTCAACGATCTGA
TTGTTCATGAGTTTGACAGCTATTGGTTCGACTTTAACTCTCAGTTCAATATTGATGCTTCCAAGCG
TGTCGGTTACAGGAACATGATTGGAGACATTCCCGCCATGATTAACCCTGTGACGACCGGCAACCCC
CTCGGCACCGGCGAATTCTTCAACCTTCCTATCCCCCTCTTTTATTCAGAGGACTCGGGTCTGGCCC
TTGCCGTTTCGGCCCTTCCCTTCAACGACATCAAGATTAACTACTGTCTCCGCAGGTGGCAGGATCT
TCTCGTCCTGAACGTGTGTGTTGGCGCCAACCCTCCCACTCTCGACGATGTTGTTCAGGTGAGCTAC
GACGCCGCCTTCGCCCTCGTCTACAGCTCGAACCCTCCCGCCATCACCAATGTCGAGACCTGGTGCC
ATTATGCCGTTGTTCACAATGACGAACGTGTCAAGATGGGTAAGAATCCCCGTGATATGGTCATTAA
GCAGGTTCAGAAGGTCAATGAGACGACCATCAACCTTTCGCAGCTCAACGCTCTCATCCCGATCGAC
ATCCGTATCTCTCACGCCGTTGTTGGCTACTTCTACGCCATCCGAAACAGCTCCACTCCCGGCGAAT
GGTCCAACTACACCACCGAGCCCGCCTATGCCGGTCTTGACCCGCTTGAGGCCGCCCAGCTTGTATA
CGAGTCGACCGCCCGTGTCAGCAACGGTTCTGACTATTACAGTCTTGTTGTGCCGTGGTACTGGCAC
AAGTCAATCCCCGAGGAGACCGGCTACCACGCGTACTCGTACTCGCTGGAGACTTTCGCCTCTGACC
CCAAGGGTTCGACCAATTATTCCAAGCTCACCAACGTGTCGAACCAGTACGTTCCTTCGACCGCTGC
CGTCAACGCCTCTGCTGGTGTGACCAACACCGGTA

>Mal_14

GGCGACCTTCTCTGACTTGGAGGCGTATCTCTATGGTGGTTGCTCGGCAGTCACCTATTTTGTGCGT
GCCATCAAAAAGGCCAATTGGTTCTCCTTCCTTCCCGTTGTTCTCCGAAATATTTCGGGTCTTCCCG
GCTTCGGTAATGAATTCTCCGCGTCTGTGAATCGCTCCGGTGATTACGTCCTCAACACCTGGCTGCG
CGTCCGTCTCCCTCTGATTGCTATTCGCCCGACCAACGCTGGTGGCGCCATCAATGCTAACGCCACC
ATCCGCTGGACCAGGAACTTTATGCACAATCTTGTTGAAAAGATTAACATCACTTTCAACGATCTGA
TTGTTCATGAGTTTGACAGCTATTGGTTCGACTTTAACTCTCAGTTCAATATTGATGCTTCCAAGCG
TGTCGGTTACAGGAACATGATTGGAGACATTCCCGCCATGATTAACCCTGTGACGACCGGCAACCCC
CTCGGCACCGGCGAATTCTTCAACCTTCCTATCCCCCTCTTTTATTCAGAGGATTCGGGTCTGGCCC
TTGCCGTTTCGGCCCTTCCCTTCAACGACATCAAGATTAACTACTGTCTCCGCAGGTGGCAGGATCT
TCTCGTCCTGAACGTGGGTGTTGGCGCCAACCCTCCCACTCTCGACGATGTTGTTCAGGTGAGCTAC
GATGCCACTTTCACCCTCATTTACAGCTCCAACCCTCCCGCCATCACCAATGTCGAGACTTGGTGCC
ATTACGCCGTCGTGCACAATGACGAACGTGTCAAGATGGGTAAGAATCCCCGTGATATGGTTATCAA
GCAGGTTCAGAAGGTCAATGAGACGACCATCAACCTTTCGCAGCTCAACGCTCTCATCCCGATTGAT
ATCCGTATCTCTCACGCCGTTGTTGGATACTTCTACGCCATCCGAAACAGCTCCACTCCCGGCGAAT
GGTCCAACTACACCACCGAGCCCGCCTATGCCGGTCTTGACCCGCTTGAGGCTGCCCAGCTTGTGTA
CGAGTCGACTGCCCGTGTCAGCAACGGTTCCGACTATTACAGCCTTGTTGTGCCGTGGTACTGGCAC
AAGTCGATCCCCGAGGAGACCGGCTACCACGCGTATTCGTACTCGCTGGAGACTTTCGCCTCTGACC
CCAAGGGTTCGACCAATTATTCCAAGCTCACCAACGTGTCGAACCAGTACGTTCCTTCGACCGCTGC
CGTCAACGCCTCTGCTGGTGTGACCAACACCGGTATT

>Mal_15

GGCGACCTTCTCTGACTTGGAGGCGTATCTTTACGGTGGTTGCTCGGCAGTCACCTATTTCGTGCGT
GCCATCAAAAAGGCCAATTGGTTCTCCTTCCTTCCCGTTGTTCTCCGAAATATTTCGGGTCTTCCCG
GCTTCGGTAATGAATTCTCCGCGTCTGTGAATCGCTCCGGTGATTACGTCCTCAACACCTGGCTGCG
TGTCCGTCTCCCTCTGATTGCCATTCGCCCGACCAACGCTGGTGGCGCCATCAATGCTAATGCCACC
ATCCGCTGGACCAGGAACTTTATGCACAATCTTGTTGAAAAGATTAACATCACTTTCAACGATCTGA
TTGTTCACGAGTTTGACAGCTACTGGTTCGACTTTAACTCTCAGTTCAATATTGATGCTTCCAAGCG
CGTCGGTTACAGGAACATGATTGGAGACATCCCCGCCATGATCAACCCTGTGACGACCGGTAACCCC
CTCGGCACCGGCGAATTCTTCAACCTTCCTATCCCCCTCTTCTATTCAGAGGACTCGGGTCTGGCTC
TTGCCGTTTCGGCCCTTCCTTTCAACGACATCAAGATTAACTACTGTCTCCGTAGGTGGCAGGATCT
TCTCGTCCTGAACGTGGGTGTTGGCGCCAACCCTCCCACTCTCGACGATGTTGTTCAGGTGAGCTAC
GATGCCACTTTCACCCTCATTTACAGCTCCAACCCTCCCGCCATCACCAATGTCGAGACTTGGTGCC
ACTACGCCGTCGTGCACAATGACGAACGTGTCAAGATGGGTAAGAATCCCCGTGATATGGTCATCAA
GCAGGTTCAGAAGGTCAATGAGACGACCATCAACCTTTCGCAGCTCAACGCTCTCATCCCGATTGAT
ATCCGTATCTCTCACGCCGTTGTTGGCTACTTCTACGCCATCCGAAACAGCTCCACTCCCGGCGAAT
GGTCCAACTACACCACCGAGCCCGCCTATGCCGGTCTTGACCCGCTTGAGGCTGCTCAGCTTGTGTA
CGAGTCGACTGCCCGTGTCAGCAACGGTTCCGACTATTACAGCCTTGTTGTGCCGTGGTACTGGCAC
AAGTCGATCCCCGAGGAGACCGGCTACCACGCGTATTCGTACTCGCTGGAGACTTTCGCCTCTGACC
CCAAGGGTTCGACCAATTATTCCAAGCTCACCAACGTGTCGAACCAGTACGTTCCTTCGACCGCTGC
CGTCAACGCCTCTGCTGGCGTGACCAACACCGGTATT

>Mal_16

GGCGACCTTTTCTGACTTGGAGGCGTATCTCTATGGTGGTTGCTCGGCAGTCACCTATTTTGTGCGT
GCCATCAAAAAGGCCAATTGGTTCTCCTTCCTTCCCGTTGTTCTCCGAAATATTTCGGGTCTTCCCG
GCTTCGGTAATGAATTCTCCGCGTCTGTGAATCGCTCCGGTGATTACGTCCTCAACACCTGGCTGCG
TGTCCGTCTCCCTCTGATTGCCATTCGCCCGACCAACGCTGGTGGCGCCATCAATGCTAATGCCACC
ATCCGCTGGACCAGGAACTTTATGCACAATCTTGTTGAAAAGATTAACATCACTTTCAACGATCTGA

TTGTTCATGAGTTTGACAGCTATTGGTTCGACTTTAACTCTCAGTTCAATATTGATGCTTCCAAGCG
TGTCGGTTACAGGAACATGATTGGAGACATTCCCGCCATGATTAACCCTGTGACGACCGGTAACCCC
CTCGGCACCGGCGAATTCTTCAACCTTCCTATCCCCCTTTTCTATTCGGAGGACTCGGGTCTGGCCC
TTGCCGTTTCGGCCCTTCCCTTCAACGACATCAAGATTAACTACTGTCTCCGCAGGTGGCAGGATCT
TCTCGTCCTGAACGTGGGTGTTGGCGCCAACCCTCCCACTCTCGACGATGTTGTTCAGGTGAGCTAC
GATGCCACTTTCACCCTCATTTACAGCTCCAACCCTCCCGCCATCACCAATGTCGAAACCTGGTGCC
ATTACGCCGTCGTGCACAATGACGAACGTGTCAAGATGGGTAAGAATCCCCGTGATATGGTTATCAA
GCAGGTTCAGAAGGTCAATGAGACGACCATCAACCTTTCGCAGCTCAACGCTCTCATCCCGATTGAT
ATCCGTATCTCTCACGCCGTTGTTGGATACTTCTACGCCATCCGAAACAGCTCCACTCCCGGCGAAT
GGTCCAACTACACCACCGAGCCCGCCTATGCCGGTCTTGACCCGCTTGAGGCTGCCCAGCTTGTGTA
CGAGTCGACTGCCCGTGTCAGCAACGGTTCCGACTATTACAGCCTTGTTGTGCCGTGGTACTGGCAC
AAGTCGATCCCCGAGGAGACCGGCTACCACGCGTACTCGTACTCGCTGGAGACTTTCGCCTCTGACC
CCAAGGGTTCGACCAATTATTCCAAGCTCACCAACGTGTCGAACCAGTACGTTCCTTCGACCGCTGC
CGTCAACGCCTCTGCTGGTGTGACCAACACCGG

>Mal_17

GACTTTCTCGGATTTGGAGGCTTACCTCTATGGTGGTTGTTCCGCCGTCACCTATTTTGTGCGTGCC
ATCAAAAAGGCCAATTGGTTCTCCTTCCTCCCTGTTGTTCTTCGTAACATCTCGGGTCTTCCCGGCT
TCGGTTCAGAGTTCTCTGCTTCTGTGAATCGTTCCGGAGATTACGTCCTCAACACCTGGCTGCGTGT
GCGTCTTCCTCTCGTGGCCATTCGTCCCACCAATACTGGTGGCGCCATCAACGCTAACGCCACCATT
CGCTGGACCAGAAACTTCATGCACAATCTTGTGGAGAAGGTTAACATCACTTTCAATGACCTCATCG
TCCATGAGTTTGACAGCTACTGGTTCGACTTCAACTCGCAGTTCAACATCGACGCTTCCAAGCGCGT
CGGTTACAGGAACATGATCGGAGATATTCCGGCCATGATTAACCCTGTGACGACCGGCAACCCTCTC
GGCACTGGCGAGTTCTTCAATCTTCCCATTCCTCTCTTCTACACCGAGGATTCCGGTCTCGCCCTTG
CTGTGTCAGCTCTTCCGTTCAACGACATCAAGATTAACTACTGTCTTCGCAGGTGGCAGGATCTGAT
TGTCCTCAACGTGGGCGTCGGCGGTAACCCTCCCACTTATGACGACATTGTCCAGGTTTCTTACGAC
TCTACTTTCACCCTCATCTACAGTTCGAACGCTCCCGCCATCACCAATGTCGAGACCTGGTGTCACT
ACGCCGTCGTCCACAACGACGAACGCGTCAAGATGGGTAAGAATCCCCGTGACATGGTCATCAAGCA
GGTGCAAAAGGTCAACGAGACGACCATCAACCTTTCACAGCTCAACGCCCTTGTCCCCATCGACATT
CGCGTGTCTCATGCCGTCGTTGGATACTTCTACGCTATCCGAAACAGTTCGACCACTGGTGAATGGT
CCAATTACACCACTGAACCAGCCTATGCCGGTCTTGACCCGCTCGAGGCCGCTCAGCTCGTGTACGA
GTCGACTGCCCGTGTCAGCAACGGTTCTGACTATTACAGCCTGATGGTGCCGTGGTATTGGCACAAG
TCGATCCCCGAGGAGACGGGTTACCACGCGTACTCTTATTCTCTCGACACCTTTGCTTCCGACCCCA
AGGGTTCGACCAATTATTCCAAGCTCACCAACGTCTCGAACCAATACGTTCCTTCGACCGCTGCTGT
CAACGCTTCTGCTGGCGTGACCAACACCGGCATTCC

>Mal_18

GGCGACCTTTTCTGACTTGGAGGCGTATCTTAGGGGGTGCTCGGCAGTCACCTATTTTGTGCGTGCC
ATCAAAAAGGCCAATTGGTTCTCCTTCCTTCCCGTTGTTCTCCGAAATATTTCGGGTCTTCCCGGCT
TCGGTAATGAATTCTCCGCGTCTGTGAATCGCTCCGGTGATTACGTCCTCAACACCTGGCTGCGTGT
CCGTCTCCCTCTGATTGCCATTCGCCCGACCAACGCTGGTGGCGCCATCAATGCTAATGCCACCATC
CGCTGGACCAGGAACTTTATGCACAATCTTGTTGAAAAGATTAACATCACTTTCAACGATCTGATTG
TTCATGAGTTTGACAGCTATTGGTTCGACTTTAACTCTCAGTTCAATATTGATGCTTCCAAGCTTGT
CGGTTACAGGAACATGATTGGAGACATTCCCGCCATGATTAACCCTGTGACGACCGGTAACCCCCTC
GGCACCGGCGAATTCTTCAACCTTCCTATCCCCCTTTTCTATTCGGAGGACTCGGGTCTGGCCCTTG
CCGTTTCGGCCCTTCCCTTCAACGACATCAAGATTAACTACTGTCTCCGCAGGTGGCAGGATCTTCT

CGTCCTGAACGTGGGTGTTGGCGCCAACCCTCCCACTCTCGACGATGTTGTTCAGGTGAGCTACGAT
GCCACTTTCACCCTCATTTACAGCTCCAACCCTCCCGCCATCACCAATGTCGAAACCTGGTGCCATT
ACGCCGTCGTGCACATGACGAACGTGTCAAGATGGGTAAGAATCCCCGTGATATGGTTATCAAGCAG
GTTCAGAAGGTCAATGAGACGACCATCAACCTTTCGCAGCTCAACGCTCTCATCCCGATTGATATCC
GTATCTCTCACGCCGTTGTTGGATACTTCTACGCCATCCGAAACAGCTCCACTCCCGGCGAATGGTC
CAACTACACCACCGAGCCCGCCTATGCCGGTCTTGACCCGCTTGAGGCTGCCCAGCTTGTGTACGAG
TCGACTGCCCGTGTCAGCAACGGTTCCGACTATTACAGCCTTGTTGTGCCGTGGTACTGGCACAAGT
CGATCCCCGAGGAGACCGGCTACCACGCGTACTCGTACTCGCTGGAGACTTTCGCCTCTGACCCCAA
GGGTTCGACCAATTATTCCAAGCTCACCAACGTGTCGAACCAGTACGTTCCTTCGACCGCTGCCGTC
AACGCCTCTGCTGGTGTGACCAACACCGG

>Mal_19

GGCGACCTTCTCTGACTTGGAGGCGTATCTTTACGGTGGTTGCTCGGCAGTCACCTATTTCGTGCGT
GCCATCAAAAAGGCCAATTGGTTCTCCTTCCTTCCCGTTGTTCTCCGAAATATTTCGGGTCTTCCCG
GATTCGGTAATGAATTCTCCGCGTCTGTGAATCGCTCCGGTGATTACGTCCTCAACACCTGGCTGCG
TGTCCGTCTCCCTCTGATTGCCATTCGCCCGACCAACGCTGGTGGCGCCATCAATGCTAATGCCACC
ATCCGCTGGACCAGGAACTTTATGCACAATCTTGTTGAAAAGGTTAACATCACTTTCAACGATCTGA
TTGTTCACGAGTTTGACAGCTATTGGTTCGACTTTAACTCTCAGTTCAATATTGATGCTTCCAAGCG
CGTCGGTTACAGGAACATGATTGGAGACATCCCCGCCATGATCAACCCTGTGACGACTGGTAACCCC
CTCGGCACCGGCGAATTCTTTAATCTTCCCATTCCCCTCTTCTATTCAGAGGACTCGGGTCTGGCTC
TTGCCGTTTCAGCCCTTCCCTTCAACGACATCAAGATTAACTACTGTCTCCGCAGGTGGCAGGATCT
TCTTGTTCTGAACGTGGGTGTTGGTGCCAACCCTCCCACTCTCGACGATGTTGTTCAGGTGAGCTAC
GATGCCGCATTCGCCCTCGTCTACAGCTCGAACCCTCCCGCCATCACCAATGTCGAGACTTGGTGCC
ACTACGCCGTCGTGCACAATGACGAACGTGTCAAGATGGGTAAGAATCCCCGTGATATGGTCATCAA
GCAGGTCCAGAAGGTCAACGAGACGACCATCAACCTTTCGCAGCTCAACGCTCTCATCCCGATTGAC
ATCCGTATCTCTCACGCCGTTGTTGGCTACTTCTACGCCATCCGAAACAGCTCCACTCCCGGCGAAT
GGTCCAACTACACCACCGAGCCCGCCTATGCCGGTCTTGACCCGCTTGAGGCTGCCCAGCTTGTATA
CGAGTCGACCGCCCGTGTCAGCAACGGTTCTGACTATTACAGTCTTGTTGTGCCGTGGTACTGGCAC
AAGTCAATCCCCGAGGAGACCGGCTACCACGCGTACTCGTACTCGCTGGAGACTTTTGCCTCTGACC
CCAAGGGTTCGACCAATTATTCCAAGCTCACCAATGTGTCGAACCAGTACGTTCCTTCGACCGCCGC
CGTCAACGCCTCTGCTGGCGTGACCAACACCGGTATT

>Mal_20

GGCGACCTTCTCCGACTTGGAGGCGTACCTTTAGAAGGGTGCTCGGCGGTCACCTATTTCGTGCGTG
CCATCAAAAAGGCTAATTGGTTCTCTTTCCTCCCCGTTGTTCTCCGAAACATTTCGGGTCTTCCCGG
TTTCGGTAACGAATTCTCCGCGTCCGTGAATCGTTCCGGCGATTACGTCCTCAACACCTGGCTTCGC
GTCCGTCTTCCTCTCATTGCTATCCGTCCGACCAATGCTGGTGGTGCCATCAACGCTAATGCCACCA
TCCGCTGGACCCGAAACTTCATGCACAATCTTGTTGAAAAGATCAACATCACCTTCAATGATCTGAT
TGTCCACGAGTTTGACAGCTACTGGTTCGACTTTAACTCCCAGTTCAACATCGATGCTTCCAAGCGC
GTCGGTTACAGGAACATGATCGGAGACATTCCCGCCATGATTAACCCCGTGACGACCGGCAACCCCC
TCGGCACCGGCGAATTCTTCAATCTTCCCATCCCCCTCTTCTATTCCGAGGACTCGGGTCTGGCTCT
TGCCGTCTCTGCCCTTCCTTTCAACGACATCAAGATCAACTACTGTCTCCGCAGGTGGCAGGATCTT
CTCGTCCTGAACGTGGGTGTTGGCGCCAACCCTCCCACTTTCGACGATGTTGTTCAGGTGAGCTACG
ACGCCACCTTCACCCTCATTTACAGCTCGAACGCTCCCGCCATCACCAATGTCGAGACTTGGTGCCA
CTACGCCGTTGTGCACAATGACGAGCGTGTCAAGATGGGTAAGAATCCCCGTGATATGGTCATCAAG
CAGGTTCAAAAGGTCAATGAGACGACCATCAATCTTTCGCAGCTCAACGCTCTCATCCCGATTGATA
TCCGTATCTCTCACGCCGTCGTTGGCTATTTCTACGCCATCAGGAACAGCTCCACTCCCGGCGAATG

GTCCAACTACACCACCGAGCCCGCCTATGCCGGTCTTGACCCGCTTGAGGCTGCCCAGCTTGTTTAC
GAGTCGACTGCCCGTGTCAGCAATGGCTCTGATTATTACAGCCTTGTTGTGCCGTGGTACTGGCACA
AGTCGATCCCCGAGGAGACCGGCTACCACGCGTACTCGTACTCTCTGGAGACTTTCGCCTCTGATCC
CAAGGGTTCGACCAATTATTCCAAGCTCACCAATGTGTCGAACCAGTACGTTCCTTCGACCGCTGCC
GTCAACGCCTCTGCTGGTGTGACCAACACCGG

>Mal_21

GGCGACCTTCTCTGACTTGGAGGCGTATCTGAGGGGGGGCTCGGCAGTCACCTATTTTGTGCGTGCC
ATCAAAAGGGCCAATTGGTTCTCCTTCCTTCCCGTTGTTCTCCGAAATATTTCGGGTCTTCCCGGCT
TCGGTAATGAATTCTCCGCGTCTGTGAATCGCTCCGGTGATTACGTCCTCAACACCTGGCTGCGCGT
CCGTCTCCCTCTGATTGCTATTCGCCCGACCAACGCTGGTGGCGCCATCAATGCTAACGCCACCATC
CGCTGGACCAGGAACTTTATGCACAATCTTGTTGAAAAGATTAACATCACTTTCAACGATCTGATTG
TTCATGAGTTTGACAGCTATTGGTTCGACTTTAACTCTCAGTTCAATATTGATGCTTCCAATTTTTG
TTGGTTACAGGAACATGATTGGAGACATTCCCGCCATGATTAACCCTGTGACGACCGGCAACCCCCT
CGGCACCGGCGAATTCTTCAACCTTCCTATCCCCCTCTTTTATTCAGAGGATTCGGGTCTGGCCCTT
GCCGTTTCGGCCCTTCCCTTCAACGACATCAAGATTAACTACTGTCTCCGCAGGTGGCAGGATCTTC
TCGTCCTGAACGTGGGTGTTGGCGCCAACCCTCCCACTCTCGACGATGTTGTTCAGGTGAGCTACGA
TGCCACTTTCACCCTCATTTACAGCTCCCACCCTCCCGCCATCACCAATGTCGAGACTTGGTGCCAT
TACGCCGTCGTGCACAATGACGAACGTGTCAAGATGGGTAAGAATCCCCGTGATATGGTTATCAAGC
AGGTTCAGAAGGTCAATGAGACGACCATCAACCTTTCGCAGCTCAACGCTCTCATCCCGATTGATAT
CCGTATCTCTCACGCCGTTGTTGGATACTTCTACGCCATCCGAAACAGCTCCACTCCCGGCGAATGG
TCCAACTACACCACCGAGCCCGCCTATGCCGGTCTTGACCCGCTTGAGGCTGCCCAGCTTGTGTACG
AGTCGACTGCCCGTGTCAGCAACGGTTCCGACTATTACAGCCTTGTTGTGCCGTGGTACTGGCACAA
GTCGATCCCCGAGGAGACCGGCTACCACGCGTATTCGTACTCGCTGGAGACTTTCGCCTCTGACCCC
AAGGGTTCGACCAATTATTCCAAGCTCACCAACGTGTCGAACCAGTACGTTCCTTCGACCGCTGCCG
TCAACGCCTCTGCTGGTGTGACCAACACCGGTATTCC

>Mal_22

GGCGACCTTCTCTGACTTGGAGGCGTATCTCTATGGTGGTTGCTCGGCAGTCACCTATTTTGTGCGT
GCCATCAAAAAGGCCAATTGGTTCTCCTTCCTTCCCGTTGTTCTCCGAAATATTTCGGGTCTTCCCG
GCTTCGGTAATGAATTCTCCGCGTCTGTGAATCGCTCCGGTGATTACGTCCTCAACACCTGGCTGCG
CGTCCGTCTCCCTCTGATTGCTATTCGCCCGACTAATGCTGGTGGTGCCATTAACGCCAACGCCACC
ATTCGCTGGACCAGGAACTTTATGCACAATCTTGTTGAAAAGATTAACATCACTTTCAACGATCTGA
TTGTTCATGAGTTTGACAGCTATTGGTTCGACTTTAACTCTCAGTTCAATATTGATGCTTCCAAGCG
TGTCGGTTACAGGAACATGATTGGAGACATTCCCGCCATGATTAACCCTGTGACGACCGGCAACCCC
CTCGGCACCGGCGAATTCTTCAACCTTCCTATCCCCCTCTTTTATTCAGAGGACTCGGGTCTGGCCC
TTGCCGTTTCGGCCCTTCCCTTCAACGACATCAAGATTAACTACTGTCTCCGCAGGTGGCAGGATCT
TCTCGTCCTGAACGTGGGTGTTGGCGCCAACCCTCCCACTCTCGACGATGTTGTTCAGGTGAGCTAC
GATGCCACTTTCACCCTCATTTACAGCTCCAACCCTCCCGCCATCACCAATGTCGAGACTTGGTGCC
ATTACGCCGTCGTGCACAATGACGAACGTGTCAAGATGGGTAAGAATCCCCGTGATATGGTTATCAA
GCAGGTTCAGAAGGTCAATGAGACGACCATCAACCTTTCGCAGCTCAACGCTCTCATCCCGATTGAT
ATCCGTATCTCTCACGCCGTTGTTGGATACTTCTACGCCATCCGAAACAGCTCCACTCCCGGCGAAT
GGTCCAACTACACCACCGAGCCCGCCTATGCCGGTCTTGACCCGCTTGAGGCTGCCCAGCTTGTGTA
CGAGTCGACTGCCCGTGTCAGCAACGGTTCCGACTATTACAGCCTTGTTGTGCCGTGGTACTGGCAC
AAGTCGATCCCCGAGGAGACCGGCTACCACGCGTATTCGTACTCGCTGGAGACTTTCGCCTCTGACC

CCAAGGGTTCGACCAATTATTCCAAGCTCACCAACGTGTCGAACCAGTACGTTCCTTCGACCGCTGC
CGTCAACGCCTCTGCTGGTGTGACCAACACCGG

>Mal_23

GGCGACCTTCTCTGACTTGGAGGCGTATCTTTACGGTGGTTGCTCGGCAGTCACCTATTTTGTGCGT
GCCATCAAAAAGGCCAATTGGTTCTCCTTCCTTCCCGTTGTTCTCCGAAATATTTCGGGTCTTCCCG
GCTTCGGTAATGAATTCTCCGCGTCTGTGAATCGCTCCGGTGATTACGTCCTCAACACCTGGCTGCG
TGTCCGTCTCCCTCTGATTGCCATTCGCCCGACCAACGCTGGTGGCGCCATCAATGCTAATGCCACC
ATCCGCTGGACCAGGAACTTTATGCACAATCTTGTTGAAAAGATTAACATCACTTTCAACGATCTGA
TTGTTCACGAGTTTGACAGCTATTGGTTCGACTTTAACTCTCAGTTCAATATTGATGCTTCCAAGCG
CGTCGGTTACAGGAACATGATTGGAGACATCCCCGCCATGATCAACCCTGTGACGACCGGTAACCCC
CTCGGCACCGGCGAATTCTTCAACCTTCCTATCCCCCTCTTTTATTCAGAGGACTCGGGTCTGGCTC
TTGCCGTTTCGGCCCTTCCTTTCAACGACATCAAGATTAACTACTGTCTCCGCAGGTGGCAGGATCT
TCTCGTCCTGAACGTGGGTGTTGGCGCCAACCCTCCCACTCTCGACGATGTTGTTCAGGTGAGCTAC
GATGCCACTTTCACCCTCATTTACAGCTCCAACCCTCCCGCCATCACCAATGTCGAGACTTGGTGCC
ACTACGCCGTTGTTCACAATGACGAGCGTGTCAAGATGGGTAAGAATCCCCGTGATATGGTTATCAA
GCAGGTTCAGAAGGTCAATGAGACGACCATCAACCTTTCGCAGCTCAACGCTCTCATTCCCATCGAC
ATCCGTATCTCTCACGCCGTTGTTGGCTACTTCTACGCCATCCGAAACAGCTCCACTCCCGGTGAAT
GGTCCAACTACACCACCGAGCCCGCTTATGCCGGTCTTGACCCGCTTGAGGCTGCCCAGCTTGTGTA
CGAGTCGACTGCCCGTGTCAGCAACGGTTCCGACTATTACAGTCTTGTTGTGCCTTGGTACTGGCAC
AAGTCGATCCCCGAGGAGACCGGCTACCACGCGTACTCGTACTCGCTGGAGACTTTCGCCTCTGACC
CCAAGGGTTCGACCAATTATTCCAAGCTCACCAACGTGTCGAACCAGTACGTTCCTTCGACCGCTGC
CGTCAACGCCTCTGCTGGTGTGACCAACACCGG

>Mi_1

TGTATTGAAGCCTTCATATACTCTGTATGTTCCTCTTCAATTCTATTTCAATCGTAACAACGGTCTT
GCCCTTCCCCTTATTGCTCTTCAATATCATCAAGTTCGAATTTACGTAAAATTCAGACAAGCTGATC
AATGTTATATCGCCAGTGATGCCTTCAAATCTGGTTGTGGCAATCTCCAACTTGATGATGTTTCTCT
CTATGTCAACTATGTTTTCCTTGACACTGAAGAACGTAGAAGATTCGCTCAAGTATCTCACGAATAT
CTTATTGAACAACTTCAATTCACTGGTGAAGAATCTGCTGGCAGTAGTAATTCTGCTAAATATAAAC
TCAATTTCAATCACCCCGTTAAAGCTATTTATTGGGTAACTAAATTGGGTAATTATCAAGGTGGTAA
ATTCATGACATACGATCCGGTTTGTTGGGAAAATGCTCGTGAAAATGCTGCCAAATTACTCCTTTTG
GCTCAATACGATCTTGACGATTGGGGTTATTTCCAAGAACCTGGAGGTTATGAATGTGAAGGTAATG
ATGGTAGAAGTTATGTTGGAGATTGTGGTGTTCAATATACAGCTGTTGATCCTTCCAATCCTTCTGA
AGAACCCAGTTATATTTTCAATGATACAACCACTGCTGAAGCTTTCGATGGTTCACTTTTAATTGGT
AAGCTTGCTCCTTGTGTTCCTCTTCTCAAGAGAAACAAGGATGTCGATCTCAAGGATAAAGTCGAAG
GAATCATCCGAATTCACACTGATTTTGAAAACGACAGAATGAAATATCCTGAAGTAGAGAAAATTAC
TCGTAATGATCTCACTCTTCACGATCTTTCAGTTCCCATTAGTAAATATGATGTTGATAACCGTGTT
GATTATATTAAGAAATTCGACGTTACTGTCTGGCAACACAACAACTTTGGTCTCCTTATTGACGGTT
CTGGTAATCCTACTCATGAAGCTGAACTTCAACTCAATGGTCAACCCCGTCAAAGTAAACGTGGTGG
TATCTGGTATGACACTGTTAATCCCACAGTTCATCATACTAAATCACCCAGAGATGGTGTCAATGTG
TTCTCATTCGCTCTTAACCCTGAAGAACATCAGCCATCTTGTACATGCAATTTCTCACGTATCGATA
CCGCGCAACTTAACTTGTGGTTCCAACATTTCACTAACCACAAATTCGCTGATGTATTCGCTGAT

>Mi_2

GATAATACTGTATTGAAGCCTTCATATACTCTGTATGTTCCTCTTCAATTCTATTTCAATCGTAACA
ACGGTCTTGCCCTTCCTCTTATTGCTCTTCAATATCATCAAGTCCGAATTTACGTAAAATTCAGACA
AGCTGATCAATGCTATATCGCCAGTGATGCCTTCAAATCTGGTTGTGGCAATCTCCAACTTGATGAT
GTTTCTCTCTATGTCAACTATGTTTTCCTTGACACTGAAGAACGTAGAAGATTCGCTCAAGTATCTC
ACGAATATCTTATTGAACAACTTCAATTCACTGGTGAAGAATCTGCTGGCAGTAGTAATTCTGCTAA
ATACAAACTCAATTTCAATCACCCCGTTAAAGCTATTTATTGGGTAACTAAATTGGGTAATTATCAA
GGTGGTAAATTCATGACATACGATCCGGTTTGTTGGGAAAATGCTCGTGAAAATGCTGCTAAATTAC
TCCTTTTGGCTCAATACGATCTTGACGATTGGGGTTATTTCCAAGAACCTGGAGGTTATGAATGTGA
AGGTAATGATGGTAGAAGTTATGTTGGAGATTGTGGTGTTCAATATACAGCTGTTGATCCTTCAAAT
CCTTCTGAAGAACCCAGTTATATTTTCAATGATACAACCACTGCTGAAGCTTTCGATGGTTCACTTT
TAATTGGTAAGCTTGCTCCTTGCGCTCCTCTTCTCAAGAGAAACAAGGATGTTGATCTTAAGGATAA
AGTTGAAGGAATCATCCGAATTCATACTGATTTTGAAAACGACAGAATGAAATATCCTGAAGTAGAG
AAAATTACTCGTAATGATCTCACTCTTCACGATCTTTCAGTTCCCATTAGTAAATATGATGTTGATA
ACCGTGTTGATTACATTAAGAAATTCGACGTTACTGTCTGGCAACACAACAACTTTGGTCTCCTTAT
TGACGGTTCTGGCAATCCTACTCATGAAGCTGAACTTCAACTCAATGGTCAACCCCGTCAAAGTAAA
CGTGGTGGTATCTGGTATGACACTGTTAATCCCACAGTTCATCATACTAAATCACCCAGAGATGGTG
TTAATGTGTTCTCATTCGCTCTTAACCCTGAAGAACATCAGCCGTCTTGTACATGCAATTTCTCACG
TATCGATACCGCGCAACTTAACTTGTGGTTCAAAGATTTCACTAACCACAAATTCGCTGA

>Mi_3

ACTACTTCTCAAGCCGTCATACACTTTATATATTCCCCTCCAATTTTATTTCTGCCGTAATAATGGT
TTAGCTCTTCCCCTTATTGCTCTTCAATATCACGTAGTCAGAATCTATGTTAAATTCAGACCCGCTG
AACAATGCTATATTGCCAGTGATGCTTTTAAATCTGGTGCCGAAAATTTCGAACTTGATGATGTTTC
AGTATATGTCAACTATGTTTATCTTGATACTGAAGAACGTAGACGTTTCGCTCAAGTTTCTCACGAA
TATCTTATTGAACAACTTCAATTTACTGGTGAAGAATCAATTGGTACCAGTAACTCAGCTAAATATA
AACTCAGTTACAATCACCCCGTTAAAGGTCTTTACTGGGTCACCAAAATGGGTAATTATCAAGGTGG
TAAATTCATGGTATATGATCATGAAGATTGGGAAAGAGCTCGTGAAAATGCCGCTAAATTAATTATC
CTCGCTCAATACGATCTTGACGAATTTGGTTATTTCAATGATGTAGCATCAGAATGTGATTCAAACA
GTTATATTGGTGATTGTGGTGTTCAATATATTGGCGTTGATCCTGCCAGTCCCGCTGAAGAACCCAC
TTATACTTTCAATGATTCACTTACCGCTGATGCTTTTGATGGATCTGTTTTAATTGGTAAACTTGCT
CCTTGTGTTCCTCTTCTTAAGAGAAACAAGGATGTTGATCTTCGTGACAAAGTAGAAGGTATTATCC
GTATTGTTACTGATTTCGACAATGACAAACTTAAATATCCTGAAGTAGAAAAGATCACCAAAAATGA
TCTTACTATTACTGATCTTTCTATCCCTGTTGACAAGTTCGATGAAGACAACCGTGTTGAATATATT
AAGAGATTCGACGTTACTGTCTGGCAACACCACAATTATGGTCTTCTTATTGACGGTACTGTAAATC
CTGTATCTGATGTAGAACTCCAACTTAATGGTCAATCACGTCAAAGCAAGAGATCTGGATTCTGGCA
TGATACTGTTGAACCTTACATGCATCACACCAGAACTCCTACTGATGGTCTCAATGTATTCTCATTT
GCTCTTAACCCTGAAGAACACCAACCTTCATGTACTTGCAATTTCTCACGTATTGATACTGCTCAAC
TCAATCTCTGGTTCAATCATTTCTCCAATAATAAATATGCTG

>Mi_4

ACTCTTCTCAAGCCGTCATACACTTTATATATTCCCCTCCAATTTTATTTCTGCCGTAATAATGGTT
TAGCTCTTCCCCTTATTGCTCTTCAATATCACGTAGTCAGAATCTACGTTAAATTCAGACCCGCTGA
ACAATGCTATATTGCCAGTGATGCTTTTAAATCTGGTGCCGAAAATTTCGAACTTGATGATGTTTCA
GTATATGTCAACTATGTTTATCTTGATACTGAAGAACGTAGACGTTTCGCTCAAGTTTCTCACGAAT

ATCTTATTGAACAACTTCAATTTACTGGTGAAGAATCAATTGGTACCAGTAACTCAGCTAAATATAA
ACTCAGTTACAATCACCCCGTTAAAGGTCTTTACTGGGTCACCAAAATGGGTAATTATCAAGGTGGT
AAATTCATGGTATATGATCATGAAGATTGGGAAAGAGCTCGTGAAAATGCCGCTAAATTAATTATCC
TCGCTCAATACGATCTTGACGAATTTGGTTATTTCAATGATGTAGCATCAGAATGTGATTCAAACAG
TTATATTGGTGATTGTGGTGTTCAATATATTGGCGTTGATCCTGCCAGTCCCGCTGAAGAACCCACT
TATACTTTCAATGATTCACTTACCGCTGATGCTTTTGATGGATCTGTTTTAATTGGTAAACTTGCTC
CTTGTGTTCCTCTTCTTAAGAGAAACAAGGATGTTGATCTTCGTGACAAAGTAGAAGGTATTATCCG
TATTGTTACTGATTTCGACAATGACAAACTTAAATATCCTGAAGTAGAAAAGATCACCAAAAATGAT
CTTACTATTACTGATCTTTCTATCCCTGTTGACAAGTTCGATGAAGACAACCGTGTTGAATATATTA
AGAGATTCGACGTTACTGTCTGGCAACACCACAATTATGGTCTTCTTATTGACGGTACTGTAAATCC
TGTATCTGATGTAGAACTCCAACTTAATGGTCAATCACGTCAAAGCAAGAGATCTGGATTCTGGCAT
GATACTGTTGAACCTTACATGCATCACACCAGAACTCCTACTGATGGTCTCAATGTATTCTCATTTG
CTCTTAACCCCGAAGAACACCAACCTTCATGTACTTGCAATTTCTCACGTATTGATACTGCTCAACT
CAATCTCTGGTTCAATCATTTCTCCAATAATAAATATGCTG

>Mi_5

TCTTCTCAAGCCGTCATACACTTTATATATTCCCCTCCAATTTTATTTCTGCCGTAATAATGGTTTA
GCTCTTCCCCTTATTGCTCTTCAATATCACGTAGTCAGAATCTACGTTAAATTCAGACCCGCTGAAC
AATGCTATATTGCCAGTGATGCTTTTAAATCTGGTGCCGAAAATTTCGAACTTGATGATGTTTCAGT
ATATGTCAACTATGTTTATCTTGATACTGAAGAACGTAGACGTTTCGCTCAAGTTTCTCACGAATAT
CTTATTGAACAACTTCAATTTACTGGTGAAGAATCAATCGGTACCAGTAACGCAGCCAAATATAAAC
TCAGTTACAATCACCCCGTTAAAGGTCTTTACTGGGTCACCAAAATGGGTAATTATCAAGGTGGTAA
ATTCATGGTATATGATCATGAAGATTGGGAAAGAGCCCGTGAAAATGCCGCTAAATTAATTATCCTC
GCTCAATACGATCTTGACGAATTTGGTTATTTCAATGATGTAGCATCAGAATGTGATTCAAACAGTT
ATATTGGTGATTGTGGTGTTCAATATATTGGCGTTGATCCTGCCAGTCCCGCTGAAGAACCCACTTA
TACTTTCAACGATTCACTTACCGCTGATGCTTTTGATGGAACTGTTTTAATTGGTAAACTTGCTCCT
TGTGTTCCTCTTCTTAAGAGAAACAAGGATGTTGATCTTCGTGACAAAGTAGAAGGTATTATCCGTA
TTGTTACTGATTTCGACAATGACAAACTTAAATATCCTGAAGTAGAAAAGATCACCAAAAATGATCT
CACTATTACTGATCTTTCTATCCCTGTTGACAAGTTCGATGAAGACAACCGTGTTGAATATATTAAG
AGATTCGACGTTACTGTCTGGCAACACCACAATTATGGTCTTCTTATTGACGGTACTGTAAATCCTG
TATCTGATGTAGAACTCCAACTTAATGGTCAATCACGTCAAAGCAAGAGATCTGGATTCTGGCATGA
TACTGTTGAACCCTACATGCATCACACCAGAACTCCTACTGATGGTCTCAATGTATTCTCATTTGCT
CTTAACCCTGAAGAACACCAACCTTCATGTACTTGCAATTTCTCACGTATTGATACTGCTCAACTCA
ATCTCTGGTTCAATCATTTCTCCAATAATAAATAT

>Mi_6

CACTCTTCTCAAGCCGTCATACACTTTATATATTCCCCTCCAATTTTATTTCTGCCGTAATAATGGT
TTAGCTCTTCCCCTTATTGCTCTTCAATATCACGTAGTCAGAATCTATGTTAAATTCAGACCCGCTG
AACAATGCTATATTGCCAGTGATGCTTTTAAATCTGGTGCCGAAAATTTCGAACTTGATGATGTTTC
AGTATATGTCAACTATGTTTATCTTGATACTGAAGAACGTAGACGTTTCGCTCAAGTTTCTCACGAA
TATCTTATTGAACAACTTCAATTTACTGGTGAAGAATCAATTGGTACCAGTAACTCAGCTAAATATA
AACTCAGTTACAATCACCCCGTTAAAGGTCTTTACTGGGTCACCAAAATGGGTAATTATCAAGGTGG
TAAATTCATGGTATATGATCATGAAGATTGGGAAAGAGCTCGTGAAAATGCCGCTAAATTAATTATC
CTCGCTCAATACGATCTTGACGAATTTGGTTATTTCAATGATGTAGCATCAGAATGTGATTCAAACA
GTTATATTGGTGATTGTGGTGTTCAATATATTGGCGTTGATCCTGCCAGTCCCGCTGAAGAACCCAC
TTATACTTTCAATGATTCACTTACCGCTGATGCTTTTGATGGATCTGTTTTAATTGGTAAACTTGCT
CCTTGTGTTCCTCTTCTTAAGAGAAACAAGGATGTTGATCTTCGTGACAAAGTAGAAGGTATTATCC

GTATTGTTACTGATTTCGACAATGACAAACTTAAATATCCTGAAGTAGAAAAGATCACCAAAAATGA
TCTTACTATTACTGATCTTTCTATCCCTGTTGACAAGTTCGATGAAGACAACCGTGTTGAATATATT
AAGAGATTCGACGTTACTGTCTGGCAACACCACAATTATGGTCTTCTTATTGACGGTACTGTAAATC
CTGTATCTGATGTAGAACTCCAACTTAATGGTCAATCACGTCAAAGCAAGAGATCTGGATTCTGGCA
TGATACTGTTGAACCTTACATGCATCACACCAGAACTCCTACTGATGGTCTCAATGTATTCTCATTT
GCTCTTAACCCTGAAGAACACCAACCTTCATGTACTTGCAATTTCTCACGTATTGATACTGCTCAAC
TCAATCTCTGGTTCAATCATTTCTCCAATAATAAATATGCTGATA

>Mi_7

TGTATTGAAGCCCTCATATACTCTGTATGTTCCTCTTCAATTTTATTTCAATCGTAATAACGGTCTT
GCCCTCCCTCTTATTGCTCTTCAATATCATCAAGTCCGAATTTACGTAAAATTCAGACAAGCTGATC
AATGCTATATTGCCAGTGATGCCTTCAAATCTGGTTGTGGTAATCTCCAACTTGATGATGTTTCTCT
CTATGTCAACTATGTTTTCCTTGACACTGAAGAACGTAGGAGATTCGCTCAAGTATCTCATGAATAT
CTTATTGAACAACTTCAATTCACTGGTGAAGAATCTGCTGGCAGTAGTAATTCTGCTAAATACAAAC
TCAATTTCAATCACCCCGTCAAAGCTATTTATTGGGTAACTAAATTGGGTAATTATCAAGGTGGTAA
ATTCATGACATACGATCCAGTTTGTTGGGAAAATGCTCGTGAAAATGCTGCTAAATTACTCCTTTTG
GCTCAATATGATCTTGACGATTGGGGTTATTTCCAAGAACCTGGAGGTTATGAATGTGAAGGTAATG
ATGGTAGAAGTTATGTTGGAGATTGTGGTGTTCAATATACAGCTGTTGATCCTTCTAATCCTTCTGA
AGAACCCAGTTATATTTTCAATGATACAACCACTGCTGAAGCTTTCGATGGTTCACTTTTAATTGGT
AAGCTCGCTCCTTGCGTTCCTCTTCTCAAGAGAAACAAGGATGTCGATCTCAAGGATAAAGTTGAAG
GAATCATTCGAATTCACACTGATTTTGAAAACGACAGAATGAAATATCCTGAAGTAGAGAAAATTAC
TCGTAATGATCTCACTCTTCATGATCTTTCAGTTCCCATTAGCAAATATGATGTTGATAACCGTGTT
GATTACATTAAGAAATTCGACGTTACTGTCTGGCAACACAACAACTTTGGTCTCCTTATTGACGGTT
CTGGTAATCCTACTCATGAAGCTGAACTTCAACTCAATGGTCAACCCCGTCAAAGTAAACGTGGTGG
TATCTGGTATGACACTGTTAATCCCACAGTTCATCACACTAAATCACCCAGAGATGGTGTCAATGTG
TTCTCATTCGCTCTTAACCCTGAAGAACATCAGCCATCTTGTACATGCAATTTCTCACGTATCGATA
CCGCGCAACTTAACTTGTGGTTCCAACATTTCACCAACCACAAATTCGCTGACGTATTCG

>Mi_8

ACTGTATTGAAGCCCTCATATACTCTGTATGTTCCTCTTCAATTTTATTTCAATCGTAATAACGGTC
TTGCCCTCCCTCTTATTGCTCTTCAATATCATCAAGTCCGAATTTACGTAAAATTCAGACAAGCTGA
TCAATGCTATATTGCCAGTGATGCCTTCAAATCTGGTTGTGGTAATCTCCAACTTGATGATGTTTCT
CTCTATGTCAACTATGTTTTCCTTGACACTGAAGAACGTAGGAGATTCGCTCAAGTATCTCATGAAT
ATCTTATTGAACAACTTCAATTCACTGGTGAAGAATCTGCTGGCAGTAGTAATTCTGCTAAATACAA
ACTCAATTTCAATCACCCCGTCAAAGCTATTTATTGGGTAACTAAATTGGGTAATTATCAAGGTGGT
AAATTCATGACATACGATCCAGTTTGTTGGGAAAATGCTCGTGAAAATGCTGCTAAATTACTCCTTT
TGGCTCAATATGATCTTGACGATTGGGGTTATTTCCAAGAACCTGGAGGTTATGAATGTGAAGGTAA
TGATGGTAGAAGTTATGTTGGAGATTGTGGTGTTCAATATACAGCTGTTGATCCTTCTAATCCTTCT
GAAGAACCCAGTTATATTTTCAATGATACAACCACTGCTGAAGCTTTCGATGGTTCACTTTTAATTG
GTAAGCTCGCTCCTTGCGTTCCTCTTCTCAAGAGAAACAAGGATGTCGATCTCAAGGATAAAGTTGA
AGGAATCATTCGAATTCACACTGATTTTGAAAACGACAGAATGAAATATCCTGAAGTAGAGAAAATT
ACTCGTAATGATCTCACTCTTCATGATCTTTCAGTTCCCATTAGCAAATATGATGTTGATAACCGTG
TTGATTACATTAAGAAATTCGACGTTACTGTCTGGCAACACAACAACTTTGGTCTCCTTATTGACGG
TTCTGGTAATCCTACTCATGAAGCTGAACTTCAACTCAATGGTCAACCCCGTCAAAGTAAACGTGGT
GGTATCTGGTATGACACTGTTAATCCCACAGTTCATCACACTAAATCACCCAGAGATGGTGTCAATG

TGTTCTCATTCGCTCTTAACCCTGAAGAACATCAGCCATCTTGTACATGCAATTTCTCACGTATCGA
TACCGCGCAACTTAACTTGTGGTTCCAACATTTCACCAACCACAAATTCGCTGACG

>Mi_9

ACTCTTCTCAAGCCGTCATACACTTTATATATTCCCCTCCAATTTTATTTCTGCCGTAATAATGGTT
TAGCTCTTCCCCTTATTGCTCTTCAATATCACGTAGTCAGAATCTACGTTAAATTCAGACCCGCTGA
ACAATGCTATATTGCCAGTGATGCTTTTAAATCTGGTGCCGAAAATTTCGAACTTGATGATGTTTCA
GTATATGTCAACTATGTTTATCTTGATACTGAAGAACGTAGACGTTTCGCTCAAGTTTCTCACGAAT
ATCTTATTGAACAACTTCAATTTACTGGTGAAGAATCAATTGGTACCAGTAACTCAGCTAAATATAA
ACTCAGTTACAATCACCCCGTTAAAGGTCTTTACTGGGTCACCAAAATGGGTAATTATCAAGGTGGT
AAATTCATGGTATATGATCATGAAGATTGGGAAAGAGCTCGTGAAAATGCCGCTAAATTAATTATCC
TCGCTCAATACGATCTTGACGAATTTGGTTATTTCAATGATGTAGCATCAGAATGTGATTCAAACAG
TTATATTGGTGATTGTGGTGTTCAATATATTGGCGTTGATCCTGCCAGTCCCGCTGAAGAACCCACT
TATACTTTCAATGATTCACTTACCGCTGATGCTTTTGATGGATCTGTTTTAATTGGTAAACTTGCTC
CTTGTGTTCCTCTTCTTAAGAGAAACAAGGATGTTGATCTTCGTGACAAAGTAGAAGGTATTATCCG
TATTGTTACTGATTTCGACAATGACAAACTTAAATATCCTGAAGTAGAAAAGATCACCAAAAATGAT
CTTACTATTACTGATCTTTCTATCCCTGTTGACAAGTTCGATGAAGACAACCGTGTTGAATATATTA
AGAGATTCGACGTTACTGTCTGGCAACACCACAATTATGGTCTTCTTATTGACGGTACTGTAAATCC
TGTATCTGATGTAGAACTCCAACTTAATGGTCAATCACGTCAAAGCAAGAGATCTGGATTCTGGCAT
GATACTGTTGAACCTTACATGCATCACACCAGAACTCCTACTGATGGTCTCAATGTATTCTCATTTG
CTCTTAACCCCGAAGAACACCAACCTTCATGTACTTGCAATTTCTCACGTATTGATACTGCTCAACT
CAATCTCTGGTTCAATCATTTCTCCAATAATAAATATGCTGATA

>Mi_10

ACTCTTCTCAAGCCGTCATACACTTTATATATTCCCCTCCAATTTTATTTCTGCCGTAATAATGGTT
TAGCTCTTCCCCTTATTGCTCTTCAATATCACGTAGTCAGAATCTACGTTAAATTCAGACCCGCTGA
ACAATGCTATATTGCCAGTGATGCTTTTAAATCTGGTGCCGAAAATTTCGAACTTGATGATGTTTCA
GTATATGTCAACTATGTTTATCTTGATACTGAAGAACGTAGACGTTTCGCTCAAGTTTCTCACGAAT
ATCTTATTGAACAACTTCAATTTACTGGTGAAGAATCAATTGGTACCAGTAACTCAGCTAAATATAA
ACTCAGTTACAATCACCCCGTTAAAGGTCTTTACTGGGTCACCAAAATGGGTAATTATCAAGGTGGT
AAATTCATGGTATATGATCATGAAGATTGGGAAAGAGCTCGTGAAAATGCCGCTAAATTAATTATCC
TCGCTCAATACGATCTTGACGAATTTGGTTATTTCAATGATGTAGCATCAGAATGTGATTCAAACAG
TTATATTGGTGATTGTGGTGTTCAATATATTGGCGTTGATCCTGCCAGTCCCGCTGAAGAACCCACT
TATACTTTCAATGATTCACTTACCGCTGATGCTTTTGATGGATCTGTTTTAATTGGTAAACTTGCTC
CTTGTGTTCCTCTTCTTAAGAGAAACAAGGATGTTGATCTTCGTGACAAAGTAGAAGGTATTATCCG
TATTGTTACTGATTTCGACAATGACAAACTTAAATATCCTGAAGTAGAAAAGATCACCAAAAATGAT
CTTACTATTACTGATCTTTCTATCCCTGTTGACAAGTTCGATGAAGACAACCGTGTTGAATATATTA
AGAGATTCGACGTTACTGTCTGGCAACACCACAATTATGGTCTTCTTATTGACGGTACTGTAAATCC
TGTATCTGATGTAGAACTCCAACTTAATGGTCAATCACGTCAAAGCAAGAGATCTGGATTCTGGCAT
GATACTGTTGAACCTTACATGCATCACACCAGAACTCCTACTGATGGTCTCAATGTATTCTCATTTG
CTCTTAACCCCGAAGAACACCAACCTTCATGTACTTGCAATTTCTCACGTATTGATACTGCTCAACT
CAATCTCTGGTTCAATCATTTCTCCAATAATAAATATGCT

>Mi_11

GCCTTCATATACTCTGTATGTTCCTCTTCAATTCTATTTCAATCGTAACAACGGTCTTGCCCTTCCT
CTTATTGCTCTTCAATATCATCAAGTCCGAATTTACGTAAAATTCAGACAAGCTGATCAATGCTATA

TCGCCAGTGATGCCTTCAAATCTGGTTGTGGCAATCTCCAACTTGATGATGTTTCTCTCTATGTCAA
CTATGTTTTCCTTGACACTGAAGAACGTAGAAGATTCGCTCAAGTATCTCACGAATATCTTATTGAA
CAACTTCAATTCACTGGTGAAGAATCTGCTGGCAGTAGTAATTCTGCTAAATACAAACTCAATTTCA
ATCACCCCGTTAAAGCTATTTATTGGGTAACTAAATTGGGTAATTATCAAGGTGGTAAATTCATGAC
ATACGATCCGGTTTGTTGGGAAAATGCTCGTGAAAATGCTGCTAAATTACTCCTTTTGGCTCAATAC
GATCTTGACGATTGGGGTTATTTCCAAGAACCTGGAGGTTATGAATGTGAAGGTAATGATGGTAGAA
GTTATGTTGGAGATTGTGGTGTTCAATATACAGCTGTTGATCCTTCAAATCCTTCTGAAGAACCCAG
TTATATTTTCAATGATACAACCACTGCTGAAGCTTTCGATGGTTCACTTTTAATTGGTAAGCTTGCT
CCTTGCGCTCCTCTTCTCAAGAGAAACAAGGATGTTGATCTTAAGAAAAAGCTTGAAGGAATCATCC
GAATTCATACTGATTTTGAAAACGACAGAATGAAATATCCTGAAGTAGAGAAAATTACTCGTAATGA
TCTCACTCTTCACGATCTTTCAGTTCCCATTAGTAAATATGATGTTGATAACCGTGTTGATTACATT
AAGAAATTCGACGTTACTGTCTGGCAACACAACAACTTTGGTCTCCTTATTGACGGTTCTGGCAATC
CTACTCATGAAGCTGAACTTCAACTCAATGGTCAACCCCGTCAAAGTAAACGTGGTGGTATCTGGTA
TGACACTGTTAATCCCACAGTTCATCATACTAAATCACCCAGAGATGGTGTTAATGTGTTCTCATTC
GCTCTTAACCCTGAAGAACATCAGCCGTCTTGTACATGCAATTTCTCACGTATCGATACCGCGCAAC
TTAACTTGTGGTTCAAAGATTTCACTAACCA

>Mi_12

CCCTCATATACTCTGTATGTTCCTCTTCAATTTTATTTCAATCGTAATAACGGTCTTGCCCTCCCTC
TTATTGCTCTTCAATATCATCAAGTCCGAATTTACGTAAAATTCAGACAAGCTGATCAATGCTATAT
TGCCAGTGATGCCTTCAAATCTGGTTGTGGTAATCTCCAACTTGATGATGTTTCTCTCTATGTCAAC
TATGTTTTCCTTGACACTGAAGAACGTAGGAGATTCGCTCAAGTATCTCATGAATATCTTATTGAAC
AACTTCAATTCACTGGTGAAGAATCTGCTGGCAGTAGTAATTCTGCTAAATACAAACTCAATTTCAA
TCACCCCGTCAAAGCTATTTATTGGGTAACTAAATTGGGTAATTATCAAGGTGGTAAATTCATGACA
TACGATCCAGTTTGTTGGGAAAATGCTCGTGAAAATGCTGCTAAATTACTCCTTTTGGCTCAATATG
ATCTTGACGATTGGGGTTATTTCCAAGAACCTGGAGGTTATGAATGTGAAGGTAATGATGGTAGAAG
TTATGTTGGAGATTGTGGTGTTCAATATACAGCTGTTGATCCTTCTAATCCTTCTGAAGAACCCAGT
TATATTTTCAATGATACAACCACTGCTGAAGCTTTCGATGGTTCACTTTTAATTGGTAAGCTCGCTC
CTTGCGTTCCTCTTCTCAAGAGAAACAAGGATGTCGATCTCAAGGATAAAGTTGAAGGAATCATTCG
AATTCACACTGATTTTGAAAACGACAGAATGAAATATCCTGAAGTAGAGAAAATTACTCGTAATGAT
CTCACTCTTCATGATCTTTCAGTTCCCATTAGCAAATATGATGTTGATAACCGTGTTGATTACATTA
AGAAATTCGACGTTACTGTCTGGCAACACAACAACTTTGGTCTCCTTATTGACGGTTCTGGTAATCC
TACTCATGAAGCTGAACTTCAACTCAATGGTCAACCCCGTCAAAGTAAACGTGGTGGTATCTGGTAT
GACACTGTTAATCCCACAGTTCATCACACTAAATCACCCAGAGATGGTGTCAATGTGTTCTCATTCG
CTCTTAACCCTGAAGAACATCAGCCATCTTGTACATGCAATTTCTCACGTATCGATACCGCGCAACT
TAACTTGTGGTTCCAACATTTCACCAACCACAAATTCGCTGACG

>Mi_13

TGATAATACTGTATTGAAGCCTTCATATACTCTGTATGTTCCTCTTCAATTCTATTTCAATCGTAAC
AATGGTCTTGCCCTTCCCCTTATTGCTCTTCAATATCATCAAGTCCGAATTTACGTAAAATTCAGAC
AAGCTGATCAATGCTATATTGCCAGTGATGCCTTCAAATCTGGTTGTGGTAATCTCCAACTTGATGA
TGTTTCTCTCTATGTTAACTATGTTTTCCTTGACACTGAAGAACGTAGGAGATTCGCTCAAGTATCT
CACGAATATCTTATTGAACAACTTCAATACACTGGCGAAGAATCTGCTGGCAGTAGTAATTCTGCTA
AATATAAACTCAATTTCAATCACCCCGTTAAAGCTATTTATTGGGTAACTAAATTGGGTAATTATCA
AGGTGGTAAATTCATGACATACGATCCGGTTTGTTGGGAAAATGCTCGTGAAAATGCTGCTAAATTA
CTCCTTTTGGCTCAATATGATCTTGACGATTGGGGTTATTTCCAAGAACCTGGAGGTTATGAATGTG

AAGGTAATGATGGTAGAAGTTATGTTGGAGATTGTGGTGTTCAATATACAGCTGTTGATCCTTCCAA
TCCTTCTGAAGAACCCAGTTATATTTTCAATGATACAACCACTGCTGAAGCTTTTGATGGTTCGCTT
TTAATTGGTAAGCTCGCTCCTTGCGTTCCTCTTCTCAAGAGAAACAAGGATGTCGATCTCAAAGATA
AAGTCGAAGGAATCATCCGAATTCACACTGATTTTGAAAACGACAGAATGAAATATCCCGAAGTAGA
GAAAATTACTCGTAATGATCTTACTCTTCATGATCTTTCAGTTCCCATTGCCAAATATGATGTTGAT
AATCGTGTTGATTATATCAAGAAATTCGACGTTACTGTCTGGCAACACAACAACTTTGGTCTCCTTA
TTGACGGTTCTGGTAATCCTACTCATGAAGCTGAGCTTCAACTCAATGGTCAACCCCGTCAAAGTAA
ACGTGGTGGTATTTGGTATGATACTGTCAATCCCACAGTTCATCACACTAAATCACCCAGAGATGGT
GTCAATGTGTTCTCATTCGCTCTTAACCCTGAAGAACATCAGCCATCTTGTACATGCAATTTCTCAC
GTATCGATACCGCGCAACTTAACTTGTGGTTCCAACATTTCACTAACCACAAATTCGCTGATGTATT
CGC

>Mi_14

CATGACAACACTCTTCTCAAGCCGTCATACACTTTATATATTCCCCTCCAATTTTATTTCTGCCGTA
ATAATGGTTTAGCTCTTCCCCTTATTGCTCTTCAATATCACGTAGTCAGAATCTATGTTAAATTCAG
ACCCGCTGAACAATGCTATATTGCCAGTGATGCTTTTAAATCTGGTGCCGAAAATTTCGAACTTGAT
GATGTTTCAGTATATGTCAACTATGTTTATCTTGATACTGAAGAACGTAGACGTTTCGCTCAAGTTT
CTCACGAATATCTTATTGAACAACTTCAATTTACTGGTGAAGAATCAATTGGTACCAGTAACTCAGC
TAAATATAAACTCAGTTACAATCACCCCGTTAAAGGTCTTTACTGGGTCACCAAAATGGGTAATTAT
CAAGGTGGTAAATTCATGGTATATGATCATGAAGATTGGGAAAGAGCTCGTGAAAATGCCGCTAAAT
TAATTATCCTCGCTCAATACGATCTTGACGAATTTGGTTATTTCAATGATGTAGCATCAGAATGTGA
TTCAAACAGTTATATTGGTGATTGTGGTGTTCAATATATTGGCGTTGATCCTGCCAGTCCCGCTGAA
GAACCCACTTATACTTTCAATGATTCACTTACCGCTGATGCTTTTGATGGATCTGTTTTAATTGGTA
AACTTGCTCCTTGTGTTCCTCTTCTTAAGAGAAACAAGGATGTTGATCTTCGTGACAAAGTAGAAGG
TATTATCCGTATTGTTACTGATTTCGACAATGACAAACTTAAATATCCTGAAGTAGAAAAGATCACC
AAAAATGATCTTACTATTACTGATCTTTCTATCCCTGTTGACAAGTTCGATGAAGACAACCGTGTTG
AATATATTAAGAGATTCGACGTTACTGTCTGGCAACACCACAATTATGGTCTTCTTATTGACGGTAC
TGTAAATCCTGTATCTGATGTAGAACTCCAACTTAATGGTCAATCACGTCAAAGCAAGAGATCTGGA
TTCTGGCATGATACTGTTGAACCTTACATGCATCACACCAGAACTCCTACTGATGGTCTCAATGTAT
TCTCATTTGCTCTTAACCCCGAAGAACACCAACCTTCATGTACTTGCAATTTCTCACGTATTGATAC
TGCTCAACTCAATCTCTGGTTCAATCATTTCTCCAATAATAAATATGCTGAT

>Mi_15

TACTGTATTGAAGCCTTCATATACTCTGTATGTTCCTCTTCAATTCTATTTCAATCGTAACAACGGT
CTTGCCCTTCCTCTTATTGCTCTTCAATATCATCAAGTCCGAATTTACGTAAAATTCAGACAAGCTG
ATCAATGCTATATCGCCAGTGATGCCTTCAAATCTGGTTGTGGCAATCTCCAACTTGATGATGTTTC
TCTCTATGTCAACTATGTTTTCCTTGACACTGAAGAACGTAGAAGATTCGCTCAAGTATCTCACGAA
TATCTTATTGAACAACTTCAATTCACTGGTGAAGAATCTGCTGGCAGTAGTAATTCTGCTAAATACA
AACTCAATTTCAATCACCCCGTTAAAGCTATTTATTGGGTAACTAAATTGGGTAATTATCAAGGTGG
TAAATTCATGACATACGATCCGGTTTGTTGGGAAAATGCTCGTGAAAATGCTGCTAAATTACTCCTT
TTGGCTCAATACGATCTTGACGATTGGGGTTATTTCCAAGAACCTGGAGGTTATGAATGTGAAGGTA
ATGATGGTAGAAGTTATGTTGGAGATTGTGGTGTTCAATATACAGCTGTTGATCCTTCAAATCCTTC
TGAAGAACCCAGTTATATTTTCAATGATACAACCACTGCTGAAGCTTTCGATGGTTCACTTTTAATT
GGTAAGCTTGCTCCTTGCGCTCCTCTTCTCAAGAGAAACAAGGATGTTGATCTTAAGGATAAAGTTG
AAGGAATCATCCGAATTCATACTGATTTTGAAAACGACAGAATGAAATATCCTGAAGTAGAGAAAAT
TACTCGTAATGATCTCACTCTTCACGATCTTTCAGTTCCCATTAGTAAATATGATGTTGATAACCGT
GTTGATTACATTAAGAAATTCGACGTTACTGTCTGGCAACACAACAACTTTGGTCTCCTTATTGACG

GTTCTGGCAATCCTACTCATGAAGCTGAACTTCAACTCAATGGTCAACCCCGTCAAAGTAAACGTGG
TGGTATCTGGTATGACACTGTTAATCCCACAGTTCATCATACTAAATCACCCAGAGATGGTGTTAAT
GTGTTCTCATTCGCTCTTAACCCTGAAGAACATCAGCCGTCTTGTACATGCAATTTCTCACGTATCG
ATACCGCGCAACTTAACTTGTGGTTCAAAGATTTCACTAACCACAAATTCGCTGATG

>Mi_16

ACTCTTCTCAAGCCGTCATACACTTTATATATTCCCCTCCAATTTTATTTCTGCCGTAATAATGGTT
TAGCTCTTCCCCTTATTGCTCTTCAATATCACGTAGTCAGAATCTATGTTAAATTCAGACCCGCTGA
ACAATGCTATATTGCCAGTGATGCTTTTAAATCTGGTGCCGAAAATTTCGAACTTGATGATGTTTCA
GTATATGTCAACTATGTTTATCTTGATACTGAAGAACGTAGACGTTTCGCTCAAGTTTCTCACGAAT
ATCTTATTGAACAACTTCAATTTACTGGTGAAGAATCAATTGGTACCAGTAACTCAGCTAAATATAA
ACTCAGTTACAATCACCCCGTTAAAGGTCTTTACTGGGTCACCAAAATGGGTAATTATCAAGGTGGT
AAATTCATGGTATATGATCATGAAGATTGGGAAAGAGCTCGTGAAAATGCCGCTAAATTAATTATCC
TCGCTCAATACGATCTTGACGAATTTGGTTATTTCAATGATGTAGCATCAGAATGTGATTCAAACAG
TTATATTGGTGATTGTGGTGTTCAATATATTGGCGTTGATCCTGCCAGTCCCGCTGAAGAACCCACT
TATACTTTCAATGATTCACTTACCGCTGATGCTTTTGATGGATCTGTTTTAATTGGTAAACTTGCTC
CTTGTGTTCCTCTTCTTAAGAGAAACAAGGATGTTGATCTTCGTGACAAAGTAGAAGGTATTATCCG
TATTGTTACTGATTTCGACAATGACAAACTTAAATATCCTGAAGTAGAAAAGATCACCAAAAATGAT
CTTACTATTACTGATCTTTCTATCCCTGTTGACAAGTTCGATGAAGACAACCGTGTTGAATATATTA
AGAGATTCGACGTTACTGTCTGGCAACACCACAATTATGGTCTTCTTATTGACGGTACTGTAAATCC
TGTATCTGATGTAGAACTCCAACTTAATGGTCAATCACGTCAAAGCAAGAGATCTGGATTCTGGCAT
GATACTGTTGAACCTTACATGCATCACACCAGAACTCCTACTGATGGTCTCAATGTATTCTCATTTG
CTCTTAACCCCGAAGAACACCAACCTTCATGTACTTGCAATTTCTCACGTATTGATACTGCTCAACT
CAATCTCTGGTTCAATCATTTCTCCAAT

>Mi_17

CTGATAATACTGTATTGAAGCCTTCATATACTCTGTATGTTCCTCTTCAATTCTATTTCAATCGTAA
CAATGGTCTTGCCCTTCCCCTTATTGCTCTTCAATATCATCAAGTCCGAATTTACGTAAAATTCAGA
CAAGCTGATCAATGCTATATTGCCAGTGATGCCTTCAAATCTGGTTGTGGTAATCTCCAACTTGATG
ATGTTTCTCTCTATGTTAACTATGTTTTCCTTGACACTGAAGAACGTAGGAGATTCGCTCAAGTATC
TCACGAATATCTTATTGAACAACTTCAATACACTGGCGAAGAATCTGCTGGCAGTAGTAATTCTGCT
AAATATAAACTCAATTTCAATCACCCCGTTAAAGCTATTTATTGGGTAACTAAATTGGGTAATTATC
AAGGTGGTAAATTCATGACATACGATCCGGTTTGTTGGGAAAATGCTCGTGAAAATGCTGCTAAATT
ACTCCTTTTGGCTCAATATGATCTTGACGATTGGGGTTATTTCCAAGAACCTGGAGGTTATGAATGT
GAAGGTAATGATGGTAGAAGTTATGTTGGAGATTGTGGTGTTCAATATACAGCTGTTGATCCTTCCA
ATCCTTCTGAAGAACCCAGTTATATTTTCAATGATACAACCACTGCTGAAGCTTTTGATGGTTCGCT
TTTAATTGGTAAGCTCGCTCCTTGCGTTCCTCTTCTCAAGAGAAACAAGGATGTCGATCTCAAAGAT
AAAGTCGAAGGAATCATCCGAATTCACACTGATTTTGAAAACGACAGAATGAAATATCCCGAAGTAG
AGAAAATTACTCGTAATGATCTTACTCTTCATGATCTTTCAGTTCCCATTGCCAAATATGATGTTGA
TAATCGTGTTGATTATATCAAGAAATTCGACGTTACTGTCTGGCAACACAACAACTTTGGTCTCCTT
ATTGACGGTTCTGGTAATCCTACTCATGAAGCTGAGCTTCAACTCAATGGTCAACCCCGTCAAAGTA
AACGTGGTGGTATTTGGTATGATACTGTCAATCCCACAGTTCATCACACTAAATCACCCAGAGATGG
TGTCAATGTGTTCTCATTCGCTCTTAACCCTGAAGAACATCAGCCATCTTGTACATGCAATTTCTCA
CGTATCGATACCGCGCAACTTAACTTGTGGTTCCAACATTTCACTAACCACAAATTCGCTGATG

>Mi_18

TGTATGTTCCTCTTCAATTCTATTTCAATCGTAACAACGGTCTTGCCCTTCCTCTTATTGCTCTTCA
ATATCATCAAGTCCGAATTTACGTAAAATTCAGACAAGCTGATCAATGCTATATCGCCAGTGATGCC
TTCAAATCTGGTTGTGGCAATCTCCAACTTGATGATGTTTCTCTCTATGTCAACTATGTTTTCCTTG
ACACTGAAGAACGTAGAAGATTCGCTCAAGTATCTCACGAATATCTTATTGAACAACTTCAATTCAC
TGGTGAAGAATCTGCTGGCAGTAGTAATTCTGCTAAATACAAACTCAATTTCAATCACCCCGTTAAA
GCTATTTATTGGGTAACTAAATTGGGTAATTATCAAGGTGGTAAATTCATGACATACGATCCGGTTT
GTTGGGAAAATGCTCGTGAAAATGCTGCTAAATTACTCCTTTTGGCTCAATACGATCTTGACGATTG
GGGTTATTTCCAAGAACCTGGAGGTTATGAATGTGAAGGTAATGATGGTAGAAGTTATGTTGGAGAT
TGTGGTGTTCAATATACAGCTGTTGATCCTTCAAATCCTTCTGAAGAACCCAGTTATATTTTCAATG
ATACAACCACTGCTGAAGCTTTCGATGGTTCACTTTTAATTGGTAAGCTTGCTCCTTGCGCTCCTCT
TCTCAAGAGAAACAAGGATGTTGATCTTAAGGATAAAGTTGAAGGAATCATCCGAATTCATACTGAT
TTTGAAAACGACAGAATGAAATATCCTGAAGTAGAGAAAATTACTCGTAATGATCTCACTCTTCACG
ATCTTTCAGTTCCCATTAGTAAATATGATGTTGATAACCGTGTTGATTACATTAAGAAATTCGACGT
TACTGTCTGGCAACACAACAACTTTGGTCTCCTTATTGACGGTTCTGGCAATCCTACTCATGAAGCT
GAACTTCAACTCAATGGTCAACCCCGTCAAAGTAAACGTGGTGGTATCTGGTATGACACTGTTAATC
CCACAGTTCATCATACTAAATCACCCAGAGATGGTGTTAATGTGTTCTCATTCGCTCTTAACCCTGA
AGAACATCAGCCGTCTTGTACATGCAATTTCTCACGTATCGATACCGCGCAACTTAACTTGTGGTTC
AAAGATTTCACTA

>Mi_19

ACTGTATTGAAGCCCTCATATACTCTGTATGTTCCTCTTCAATTTTATTTCAATCGTAATAACGGTC
TTGCCCTCCCTCTTATTGCTCTTCAATATCATCAAGTCCGAATTTACGTAAAATTCAGACAAGCTGA
TCAATGCTATATTGCCAGTGATGCCTTCAAATCTGGTTGTGGTAATCTCCAACTTGATGATGTTTCT
CTCTATGTCAACTATGTTTTCCTTGACACTGAAGAACGTAGGAGATTCGCTCAAGTATCTCATGAAT
ATCTTATTGAACAACTTCAATTCACTGGTGAAGAATCTGCTGGCAGTAGTAATTCTGCTAAATACAA
ACTCAATTTCAATCACCCCGTCAAAGCTATTTATTGGGTAACTAAATTGGGTAATTATCAAGGTGGT
AAATTCATGACATACGATCCAGTTTGTTGGGAAAATGCTCGTGAAAATGCTGCTAAATTACTCCTTT
TGGCTCAATATGATCTTGACGATTGGGGTTATTTCCAAGAACCTGGAGGTTATGAATGTGAAGGTAA
TGATGGTAGAAGTTATGTTGGAGATTGTGGTGTTCAATATACAGCTGTTGATCCTTCTAATCCTTCT
GAAGAACCCAGTTATATTTTCAATGATACAACCACTGCTGAAGCTTTCGATGGTTCACTTTTAATTG
GTAAGCTCGCTCCTTGCGTTCCTCTTCTCAAGAGAAACAAGGATGTCGATCTCAAGGATAAAGTTGA
AGGAATCATTCGAATTCACACTGATTTTGAAAACGACAGAATGAAATATCCTGAAGTAGAGAAAATT
ACTCGTAATGATCTCACTCTTCATGATCTTTCAGTTCCCATTAGCAAATATGATGTTGATAACCGTG
TTGATTACATTAAGAAATTCGACGTTACTGTCTGGCAACACAACAACTTTGGTCTCCTTATTGACGG
TTCTGGTAATCCTACTCATGAAGCTGAACTTCAACTCAATGGTCAACCCCGTCAAAGTAAACGTGGT
GGTATCTGGTATGACACTGTTAATCCCACAGTTCATCACACTAAATCACCCAGAGATGGTGTCAATG
TGTTCTCATTCGCTCTTAACCCTGAAGAACATCAGCCATCTTGTACATGCAATTTCTCACGTATCGA
TACCGCGCAACTTAACTTGTGGTTCCAAAATTTCACCAACCACAAATTCGCTGACG

>Mi_20

TTCCTGATAATACTGTATTGAAGCCTTCATATACTCTGTATGTTCCTCTTCAATTCTATTTCAATCG
TAACAATGGTCTTGCCCTTCCCCTTATTGCTCTTCAATATCATCAAGTCCGAATTTACGTAAAATTC
AGACAAGCTGATCAATGCTATATTGCCAGTGATGCCTTCAAATCTGGTTGTGGTAATCTCCAACTTG
ATGATGTTTCTCTCTATGTCAACTATGTTTTCCTTGACACTGAAGAACGTAGGAGATTCGCTCAAGT
ATCTCACGAATATCTTATTGAACAACTTCAATTCACTGGCGAAGAATCTGCTGGCAGTAGTAATTCT
GCTAAATATAAACTCAATTTCAATCACCCCGTTAAAGCTATTTATTGGGTAACTAAATTGGGTAATT
ATCAAGGTGGTAAATTCATGACATACGATCCGGTTTGTTGGGAAAATGCTCGTGAAAATGCTGCTAA

ATTACTCCTTTTGGCTCAATATGATCTTGATGATTGGGGTTATTTCCAAGAACCTGGAGGTTATGAA
TGTGAAGGTAATGATGGTAGAAGTTATGTTGGAGATTGTGGTGTTCAATATACAGCTGTTGATCCTT
CCAATCCTTCTGAAGAACCCAGTTATATTTTCAATGATACAACCACTGCTGAAGCTTTTGATGGTTC
GCTTTTAATTGGTAAGCTCGCTCCTTGCGTTCCTCTTCTCAAGAGAAACAAGGATGTCGATCTCAAA
GATAAAGTCGAAGGAATCATCCGAATTCACACTGATTTTGAAAACGACAGAATGAAATATCCCGAAG
TAGAGAAAATTACTCGTAATGATCTTACTCTTCATGATCTTTCAGTTCCCATTGCCAAATATGATGT
TGATAATCGTGTTGATTATATCAAGAAATTCGACGTTACTGTCTGGCAACACAACAACTTTGGTCTC
CTTATTGACGGTTCTGGTAATCCTACTCATGAAGCTGAACTTCAACTCAATGGTCAACCCCGTCAAA
GTAAACGTGGTGGTATTTGGTATGATACTGTCAATCCCACAGTTCATCACACTAAATCACCCAGAGA
TGGTGTCAATGTGTTCTCATTCGCTCTTAACCCTGAAGAACATCAGCCATCTTGTACATGCAATTTC
TCACGTATCGATACCGCGCAACTTAACTTGTGGTTCCAACATTTCACTAACCACAAATTCGCTGAT

>Mi_21

ACTCTTCTCAAGCCGTCATACACTTTATATATTCCCCTCCAATTTTATTTCTGCCGTAATAATGGTT
TAGCTCTTCCCCTTATTGCTCTTCAATATCACGTAGTCAGAATCTATGTTAAATTCAGACCCGCTGA
ACAATGCTATATTGCCAGTGATGCTTTTAAATCTGGTGCCGAAAATTTCGAACTTGATGATGTTTCA
GTATATGTCAACTATGTTTATCTTGATACTGAAGAACGTAGACGTTTCGCTCAAGTTTCTCACGAAT
ATCTTATTGAACAACTTCAATTTACTGGTGAAGAATCAATTGGTACCAGTAACTCAGCTAAATATAA
ACTCAGTTACAATCACCCCGTTAAAGGTCTTTACTGGGTCACCAAAATGGGTAATTATCAAGGTGGT
AAATTCATGGTATATGATCATGAAGATTGGGAAAGAGCTCGTGAAAATGCCGCTAAATTAATTATCC
TCGCTCAATACGATCTTGACGAATTTGGTTATTTCAATGATGTAGCATCAGAATGTGATTCAAACAG
TTATATTGGTGATTGTGGTGTTCAATATATTGGCGTTGATCCTGCCAGTCCCGCTGAAGAACCCACT
TATACTTTCAATGATTCACTTACCGCTGATGCTTTTGATGGATCTGTTTTAATTGGTAAACTTGCTC
CTTGTGTTCCTCTTCTTAAGAGAAACAAGGATGTTGATCTTCGTGACAAAGTAGAAGGTATTATCCG
TATTGTTACTGATTTCGACAATGACAAACTTAAATATCCTGAAGTAGAAAAGATCACCAAAAATGAT
CTTACTATTACTGATCTTTCTATCCCTGTTGACAAGTTCGATGAAGACAACCGTGTTGAATATATTA
AGAGATTCGACGTTACTGTCTGGCAACACCACAATTATGGTCTTCTTATTGACGGTACTGTAAATCC
TGTATCTGATGTAGAACTCCAACTTAATGGTCAATCACGTCAAAGCAAGAGATCTGGATTCTGGCAT
GATACTGTTGAACCTTACATGCATCACACCAGAACTCCTACTGATGGTCTCAATGTATTCTCATTTG
CTCTTAACCCTGAAGAACACCAACCTTCATGTACTTGCAATTTCTCACGTATTGATACTGCTCAACT
CAATCTCTGGTTCAATCATTTCTCCAATAATAAATATGCTG

>Mi_22

ACTGTATTGAAGCCTTCATATACTCTGTATGTTCCTCTTCAATTCTATTTCAATCGTAACAACGGTC
TTGCCCTTCCTCTTATTGCTCTTCAATATCATCAAGTCCGAATTTACGTAAAATTCAGACAAGCTGA
TCAATGCTATATCGCCAGTGATGCCTTCAAATCTGGTTGTGGCAATCTCCAACTTGATGATGTTTCT
CTCTATGTCAACTATGTTTTCCTTGACACTGAAGAACGTAGAAGATTCGCTCAAGTATCTCACGAAT
ATCTTATTGAACAACTTCAATTCACTGGTGAAGAATCTGCTGGCAGTAGTAATTCTGCTAAATACAA
ACTCAATTTCAATCACCCCGTTAAAGCTATTTATTGGGTAACTAAATTGGGTAATTATCAAGGTGGT
AAATTCATGACATACGATCCGGTTTGTTGGGAAAATGCTCGTGAAAATGCTGCTAAATTACTCCTTT
TGGCTCAATACGATCTTGACGATTGGGGTTATTTCCAAGAACCTGGAGGTTATGAATGTGAAGGTAA
TGATGGTAGAAGTTATGTTGGAGATTGTGGTGTTCAATATACAGCTGTTGATCCTTCAAATCCTTCT
GAAGAACCCAGTTATATTTTCAATGATACAACCACTGCTGAAGCTTTCGATGGTTCACTTTTAATTG
GTAAGCTTGCTCCTTGCGCTCCTCTTCTCAAGAGAAACAAGGATGTTGATCTTAAGGATAAAGTTGA
AGGAATCATCCGAATTCATACTGATTTTGAAAACGACAGAATGAAATATCCTGAAGTAGAGAAAATT
ACTCGTAATGATCTCACTCTTCACGATCTTTCAGTTCCCATTAGTAAATATGATGTTGATAACCGTG

TTGATTATATTAAGAAATTCGACGTTACTGTCTGGCAACACAACAACTTTGGTCTCCTTATTGACGG
TTCTGGTAATCCTACTCATGAAGCTGAACTTCAACTCAATGGTCAACCCCGTCAAAGTAAACGTGGT
GGTATCTGGTATGACACTGTTAATCCCACAGTTCATCATACTAAATCACCCAGAGATGGTGTCAATG
TGTTCTCATTCGCTCTTAACCCTGAAGAACATCAGCCATCTTGTACATGCAATTTCTCACGTATCGA
TACCGCGCAACTTAACTTGTGGTTCAAAGATTTCACTAACCACAAATTCGCTGAT

>Mi_23

ACTCTTCTCAAGCCGTCATACACTTTATATATTCCCCTCCAATTTTATTTCTGCCGTAATAATGGTT
TAGCTCTTCCCCTTATTGCTCTTCAATATCACGTAGTCAGAATCTACGTTAAATTCAGACCCGCTGA
ACAATGCTATATTGCCAGTGATGCTTTTAAATCTGGTGCCGAAAATTTCGAACTTGATGATGTTTCA
GTATATGTCAACTATGTTTATCTTGATACTGAAGAACGTAGACGTTTCGCTCAAGTTTCTCACGAAT
ATCTTATTGAACAACTTCAATTTACTGGTGAAGAATCAATTGGTACCAGTAACTCAGCTAAATATAA
ACTCAGTTACAATCACCCCGTTAAAGGTCTTTACTGGGTCACCAAAATGGGTAATTATCAAGGTGGT
AAATTCATGGTATATGATCATGAAGATTGGGAAAGAGCTCGTGAAAATGCCGCTAAATTAATTATCC
TCGCTCAATACGATCTTGACGAATTTGGTTATTTCAATGATGTAGCATCAGAATGTGATTCAAACAG
TTATATTGGTGATTGTGGTGTTCAATATATTGGCGTTGATCCTGCCAGTCCCGCTGAAGAACCCACT
TATACTTTCAATGATTCACTTACCGCTGATGCTTTTGATGGATCTGTTTTAATTGGTAAACTTGCTC
CTTGTGTTCCTCTTCTTAAGAGAAACAAGGATGTTGATCTTCGTGACAAAGTAGAAGGTATTATCCG
TATTGTTACTGATTTCGACAATGACAAACTTAAATATCCTGAAGTAGAAAAGATCACCAAAAATGAT
CTTACTATTACTGATCTTTCTATCCCTGTTGACAAGTTCGATGAAGACAACCGTGTTGAATATATTA
AGAGATTCGACGTTACTGTCTGGCAACACCACAATTATGGTCTTCTTATTGACGGTACTGTAAATCC
TGTATCTGATGTAGAACTCCAACTTAATGGTCAATCACGTCAAAGCAAGAGATCTGGATTCTGGCAT
GATACTGTTGAACCTTACATGCATCACACCAGAACTCCTACTGATGGTCTCAATGTATTCTCATTTG
CTCTTAACCCCGAAGAACACCAACCTTCATGTACTTGCAATTTCTCACGTATTGATACTGCTCAACT
CAATCTCTGGTTCAATCATTTCTCCAATAATAAATATGCTGAT

>Mi_24

CTTTTAAAACCTTCATACACACTTTTTATTCCTCTTCAATTTTATTTCTGCCGCAATAATGGTCTTG
CTCTTCCTCTTATTGCTCTTCAATATCACATTGTTAAATTCCACGTTAAATTCAGGCCCGTAGAACA
ATGCTACATTGCTACTGATGCTTTCAAAGCTGGATCTAACAATCCTGAAATTGATGATGTTTCAGTA
TATGTAAATTATGTTTATCTTGATACTGAAGAACGTAGGAGATTTGCTCAAGTGTCTCACGAATATC
TTATCGAACAACTTCAATTTACTGGAGAAGAATCCGCAGGAAATTCTAATTCCGCTAAATATAAACT
CAATTTCAATCACCCTGTCAAAGCTCTTTATTGGGTCACTAAATTAGGTAATTATCAAGGAGGTAAA
TTCATGGCATATGATCCTACCAATTGGGAAACTGCCCGTGAAAATGCCGCTAAATTACTCCTTTTAT
CACAATATGATCTTGACGAATATGGTTTCTTCAATGATGTTGGAATTGAAAATAATAGTAACAATTA
CGTTGGTGATTGTGGTGTTCAATATGTAGCTGTTGACCCTGCCAGTCACAGTGAAGAACCCACTTAT
ATTTTCAATGATTCCAATACCGCTGAAAAATTCAATGGTTCCACTCTCATTGGTAAACTTGCTTCAT
GTGTTCCTCTTCTTAAGAGAACTAAAGATAATGATCTCAGAGAAAAAGTAGAAGGTATAATTCGTAT
TCATCACGATGAAAATAATGGTAATTATCCTGAAGTTGAAAAAGTCACTAAAAATGACTTAACAATT
CATGATCTTTCAGTTCCTATTAATAAATTCAATGAAGATAATCGTAATGAATATATCAAACGTTATG
ACATTACTGTATGGCAACACCACAATCATGGTCTCCTCATTGACGGATCCATCAATCCTACCAGCGA
AGCTGAACTTCAACTTAATGGTCAAGCTCGTCAAAGCAAGAGATCTGGTTCATGGTACGATACAGTT
TCTCCCAAGATGCATCACACTAGAACTCCCAATGATGGTCTTAATGTTTTCTCATTCGCAATTAACC
CTGAAGAACACCAACCTTCATGTACTTGCAATTTCTCACGAATTGATACTGCTCAACTCAATTTATG
GTTCAATCATTTTGCTAACAATAAGTTTGCTG

>Pan_1

CCAAACCTCGCACCACTTTGGCGCCGTGCGCGTCCACCGCCAAGCCGATCGTCTACGCGACAATGTC
GACGACGCGCGTTGGGGCGTGTTTGTCATGAAACCGACAACCGGCGAGACTCCTGTCCATGACGGCG
GTCTGTCTGGAGATGACGGTGCAACCGCAGTCCATGCTGGCGCCATTGACCACATGGAAGAGGACCT
TGCGAACCGTCAGGTGTATGTCATGCAGTGCGACACTGAACTAGAGGCCATTGAAGGATGGCGCGAT
CTCATCGTGCTCGACGTTCAGCCGAGCGTCGTCGAGGGCTACAATACGGATGCCTTTGATTTTGGCT
GGCTGGGCGTGCGTGCCGAGGTGTGTGCCGGCTATGGCATAAGGTCGCGCCTCTTTGAAGCCGGCGT
GCTCATCGGCGAGCACACGCCGATGCGTCGCAAGGATCTCGACTCGGCAGCCAAGGGCTCCAACACG
CTCAATTTCATCCCCATGCCGGGACGCATCCTCATCGATATGTACCACATTGTCAAGGCCGAAAGAC
GCCTCGAATCGTACACGCTCGAAA

>Pan_2

CCAAACCTCGCACCACTTTGGCGCCGTGCGCGTCCACCGGCCGGTCGAGGCCGAGCGCGACCGCGTC
GACGACCCCAAGCAGCGCGAGCGCTGGGGGGTACGCGAGATGGATCTCACCTCGGCCGGTCGGCCGG
AAGCAACAGCGACGACGGCGGGCCACATGGTGGACGCTGAAGAGGAGCCGTCTCAGCGCGAGGTCTA
TGTGATGCAGTGCGCCACCGAGTTGGAGGCCATCGAGGGGTGGCGCGACCTCATCGTGCTCGACGTG
CAGCCCAGCGTCGTCGAGGGCTACAACACGGACGCCTTTGATTTCGGCTGGCTGGGCGTGCGCGCCG
AGCGCTGCGCCCGGTACGGCGTGCGCTCGCGCCTCTTTGAGGCCGGCGTGCTCATCGGCGAGCACAC
GCCCATGCGCCGCAAGGACCTGGACTCGGCCGCCAAGGGCTCCAACACGCTCAACTTTATCCCGATG
CCGGGGCGCATCCTCGTCGACATGTACCATATCGTCAAGGCCGAGAAGCGTCTCGAATCGTACACGC
TCGAA

>Pan_4

GAGGCCGAGCGCGACCGCGTCGACGACCCCAAGCAGCGCGAGCGCTGGGGCGTGCTTGAAATGGGCA
TGGCGTCGGCCGATCGGCCAGAAGCAGCAACGGCTGCGGCTGCGACGACGACGGACCACACGGTGGA
CGCCGGGCGGGAGCGGTCCCAGCGCGAGGTCTATGTGATGCAGTGCGCCACCGAGTTGGAGGCCATC
GAGGGGTGGCGCGACCTCATCGTGCTCGACGTGCAGCCCAGCGTCGTCGAGGGCTACAACACGGACG
CCTTTGATTTCGGCTGGCTGGGCGTGCGCGCCGAGCGCTGCGCCCGGTACGGCGTGCGCTCGCGCCT
CTTTGAGGCCGGCGTGCTCATCGGCGAGCACACGCCCATGCGCCGCAAGGACCTGGACTCGGCCGCC
AAGGGCTCCAACACGCTCAACTTTATCCCGATGCCGGGGCGCATCCTCGTCGACATGTACCATATCG
TCAAGGCCGAGAAGCGTCTCGAATCGTACACGCTCGA

>Pan_5

GACCGGGTGCGCGATGCCGTGGACGATCCGCGGTGGGGCGTGTTTGTCATGAAACCCAACAGCAAGG
CGTCTCTCGACAGCGACGGTCTGTCCGGGGGCGGGGGCGTCAATGACAATGGCAACGTCAATGGCAA
CGGTAGTGATAATGGCACAATGACGAACAGCGTCGCGGCTGTCGCCACCCACGAGGAAGAGAGTCTC
GTCGACCGCCAGGTGTACGTGATGCAATGCGCCACAGAATTAGAGGCCATCGAGGGGTGGCGCGATC
TCATCGTGCTCGACGTACAGCCGAGCGTCGTCGAGGGCTACAACACGGACGCCTTTGATTTCGGCTG
GTTGGGCGTGCGCGCCCAAGTGTGCGCCGGCTATGGGATCAGGTCGCGCCTCTTTGAGGCGGGCGTG
CTCATCGGCGAGCACACGCCGATGCGTCGCAAGGACCTCGACTCGGCGGCCAAGGGTTCCAACACGC
TCAACTTTATCCCCATGCCGGGACGTATCCTCATCGACA

>Pan_6

GAGCGCGATCGCGTCGGCGACCCCAAGCAGCGCGAGCGCTGGGGCGTGCTCGAAATGGACCGCACTT
TGGCCGATCGGTCCGAAGCAACAACAACGACGGCGACGGACCGCACGGCGGACGACGCGCGGGAGCG
TTCTCAGCGCGAGGTCTATGTGATGCAGTGCGCCACCGAGTTGGAGGCCATCGAGGGGTGGCGCGAT
CTCATCGTGCTCGACGTGCAGCCCAGCGTCGTCGAGGGCTACAACACGGACGCCTTTGATTTCGGCT
GGCTGGGCGTGCGCGCCGAGCGCTGCGCCCGGTACGGCGTGCGCTCGCGCCTCTTTGAGGCCGGCGT
GCTCATCGGCGAGCACACGCCCATGCGCCGCAAGGACCTGGACTCGGCCGCCAAGGGCTCCAACACG
CTCAACTTTATCCCGATGCCGGGGCGCATCCTCGTCGACATGTACCACATCGTCAAGGCCGAGAAGC
GTCTCGAATCGTACACGCTCGAA

>Pan_3

CTGCGATGCTCCACCCTCTGTCGCCCGCCGATCGCGGCGCCGTCACACTGGTGGCCGACTACCTCGG
GCTCGACCTCGGCGCGACGACGTGCCTCGCCGGCGACGTGCTGCGTTACAGGGTGGCGATGGCGCCC
GACTCGGTGCGTCTCATGATCTTGGCAATTTCGCCCTCGGCCTTTTGGTCCGATCGTATCGGTCTGT
GCGACTGGAGCGCAAACGGCGCCAAGGCCACCTACGTCCCGGCGATGCGCTCGTGGACGCTGGCCGA
GGCTCGCGACATGCTGTCACTGGCGCTCAACATACCGGCCGATCTCATGGTCGCACACTTTTGTCTG
ACGCGTCGTAACGGCACAACGCGGCCCAATGCGCGCCTGCCGCTCGATTCGACAACCGTGCGTCTCC
GAGACGTGCACGCCGCCGGGCGCTCGCCGTTGGCCCTTCTCATCCATCAAAAGGCCTTTCTGGCCAA
CGTGGATGCCCTCCCGCTGTCGAGCCTCTCTCTCCCTGGGCCGCAACAGCCGACGACCAACGGCGAC
GCCGATGCATCGCCGCAGACACCAAAGAGCAGGCCCCGCTCTTTGTTAGAGACAATGATACAACCGC
CCGACGGGCTTGTGCTCATCTTTGTGAGATGCTTTGACCGAAGCAAGGAATCGCTGTCCAGAGCGCA
GCCCCTTCTCGTCGACGTCCGAGACACGATCGCCGCGGTTGTGCCGGCCATGTGCGCCCTGCTCTCC
TTGTCTGGAAGCCGCCATGTGCACCTCTTTGAGGAGGTGTCGATGTACATGGTGCTGGAAGTGGATC
CGACGTTGGC

>Ced_1

TTCCTCCTCTAGCCTTGAGTGAATTTAGGGACCTAGTCTACGAAGCAGGAGATGTAACTTTGCTTGA
GCACTACCGTCTGCCTTGTTCTTCACACAACCTTCAAACCATTGTGGAGAGAGGTCAACAGAGTACG
ATCAAACACCTGGTGAACAAAGGTTACACCTCTGCTCTTCTCTATGAAGCTTGTAGACAGAACCAGG
TAGATCTGGCAAAGAGAATGTACAGAGCAAAGTCAGAGTTGTGTCTTTGTACGGGAGAAGCTGTGCA
GAATGCTGCCTTGGATGTTCTCTCTTGGATAGTAGCAAGAGATCCATCTTACTATAATCTCGTTCTA
CATGCAGCTCTGCATCACCACAAGGTAGAGGTGCTAAGATGGCTTCCTCAAAGACAACTAAGAAAGA
TGAGCAAGAGAGATTTGTTTGTTAGAGCTTGCTCAGCTCCCAGTATGGACCTGTTTAACTTTTTGTT
GGAGATGAACTACATTAGTAAACAAACCATAACTCTCTCCCTCAGGTTAGCAGGAAGTCTTCATGTG
CAGATGCTGAGAACCTTGGTAAAGGAGAGAGGCTGGGTTCTGTGTGAAGAAATGTTTGATGCTGCTT
TGGAGCTGGGTTGTGAACAGATGCTATCCTGTTTGTTGGAACTAGAT

>Pit_1

TCCAACAGGAGCAAATGGAAAAGATGGCTGCACCGGTCAAACAGGAGCCTCGGGGACTAACGGTAAA
GATGGGTGTACTGGGCCAACGGGAGCCAATGGAAGGGATGGATTTATTGGTTACACTGGACCGACAG
GTAGCCAAGGTCCAACTGGACCTGCCGGAAGAGATGGGTTTACAGGTTACACTGGACCGACAGGTAG
CCAAGGTTTAGTTGGGCCAACGGGAGCTAGTGGAAGAGATGGAGTGAATGGAATCGATGGTTTCACT
GGCCCAACTGGTCCTGCTGGAAGAGATGGAGTGAATGGAATCGATGGATCGATCGGCCCAACTGGAC
CTGCTGGAAGAGACGGAAATGACGGATCAATCGGCCCAACTGGTCCCGCTGGAGTGGCAGGAATCAC

ATTGATTTCTGGAGTTTGGACTCCTGAATTCTTACAAGTTGTCGGATTTACTGGTTTCACTGGTCTA
GAAGGAAGTTATATGAGAATTGGCAATTTCGTTATGGCGAATTGGTCGATGACAGTCTTCCCCGAAG
CGACTGAAGGAGTGTTGAACGCAACCTTCAAGCCACCAATTTTCTTCGGCGGACCTTATGAGTTGAG
CAGT
